# Controlling the formation and alignment of low molecular weight gel ‘noodles’†

**DOI:** 10.1039/d1cc03378f

**Published:** 2021-08-04

**Authors:** Daniel McDowall, Matthew Walker, Massimo Vassalli, Marco Cantini, Nikul Khunti, Charlotte Jennifer Chante Edwards-Gayle, Nathan Cowieson, Dave J. Adams

**Affiliations:** Joseph Black Building, University Place Glasgow G12 8QQ UK dave.adams@glasgow.ac.uk; Centre for the Cellular Microenvironment, University of Glasgow G12 8LT UK; Diamond Light Source Ltd, Harwell Science and Innovation Campus Didcot OX11 0QX UK

## Abstract

We show how to control the formation and alignment of gel ‘noodles’. Nanostructure alignment can be achieved reproducibly by extensional deformation as the filaments form. Using a spinning technique, very long and highly aligned filaments can be made. The Young's moduli of the gel noodles are similar to that of a bulk gel. By using two syringe pumps in a concentric flow setup, we show that a filament-in-filament morphology can be created.

## Abstract 2

Extensional deformations induce nanostructure alignment in low molecular weight gel noodles during injection.

## Graphical abstract

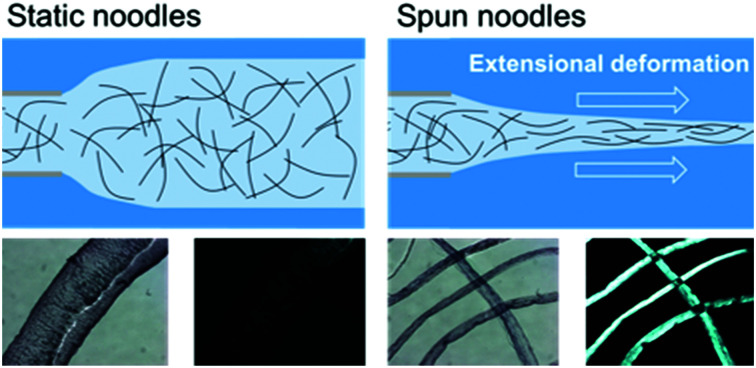

Low molecular weight gels (LMWGs) are formed from small molecules that self-assemble into 1D structures that create an entangled network and so form a gel.^1,2^ The 1D structures in a gel typically adopt a random arrangement but the 1D structures can be aligned by methods such as magnetic fields,^3–5^ electric fields^6^ and shear.^6,7^ Nanostructure alignment has potential applications from cell culturing to organic electronics.^8–10^ Low molecular weight gel filaments (or ‘noodles’^11^) were first reported in 2010 by Zhang and co-workers working with a peptide amphiphile solution.^11^ The gel filaments were formed by manually dispensing the self-assembled amphiphile solution from a pipette into a CaCl_2_ trigger solution. Initially, the pre-gel solution formed unaligned filaments when extruded. A thermal annealing process of the pre-gel solution changed the self-assembled structures and lead to an increase in viscosity; when this solution was used, aligned filaments were formed. Other work using different materials did not require a pre-extrusion heat/cool to form aligned gel filaments.^10^ Many researchers have used this technique to form self-assembled gel noodles with a wide range of different compounds.^10,12–18^ Clearly, the exact nature of the self-assembled structures present in the pre-gel solution (such as morphology, concentration and surface charge) are crucial to aligned filament formation.

Most researchers form gel filaments using injection from a pipette or a needle. This approach is effective and easy but has limitations because injection speed cannot be accurately measured and reproduced. The filament morphology and nanostructure alignment will depend on many factors including injection flow rate, pipette/needle inner diameter, the gel trigger medium and how the injection is performed (*e.g.* static or dragged) among others. These factors are yet to be investigated in detail. Here, we describe the conditions required to form gel noodles and then how to control the morphology and alignment of the noodles, linking this with their mechanical properties. We extend this to show how a noodle-in-noodle morphology can be accessed.

A functionalised dipeptide, 1ThNapFF (Fig. 1a) was selected as the focus of this study. 1ThNapFF self-assembles into worm-like micelles at high pH forming shear-thinning solutions.^19^ Like the peptide-amphiphile mentioned above, 1ThNapFF forms strong gels with calcium salts.^19^ For noodle formation, 50 mM CaCl_2_ was chosen as the gel trigger. 1ThNapFF solutions from 1–20 mg mL^−1^ (at pH 11.3) were studied by shear rheology and then tested for the ability to form gel filaments. All solutions at concentrations of 2 mg mL^−1^ and above showed shear-thinning behaviour (Section S2.1, ESI†) and formed gel noodles. For the 2 mg mL^−1^ samples, the gel filaments were difficult to see so 80 ppm Nile Blue A was added. The self-assembled structures present in the 1ThNapFF pre-gel solutions were probed using small angle X-ray scattering (SAXS) over a concentration range of 1 mg mL^−1^ to 15 mg mL^−1^ (Section S2.2, ESI†). Small angle scattering techniques has previously been extensively used to study self-assembled rod-like structures in aqueous solution.^20,21^ The data at 1.0 and 1.5 mg mL^−1^ show isotropic, low scattering intensity. These data were not fitted to any structural models and correlate with either no or a very low concentration of self-assembled structures. At concentrations of 2 mg mL^−1^ and greater, the samples scatter strongly and were fitted to either the cylinder or flexible cylinder models. These data depict long cylindrical structures with radii of around 2.1 nm at all concentrations of ≥2 mg mL^−1^, suggesting no significant change in the fibre cross-sections. From a concentration of 5 mg mL^−1^ and above, the addition of a flexible component to the fitting model was required. The rheology and SAXS data show that solutions with self-assembled 1D structures and therefore higher viscosities are required for effective noodle formation. The low viscosity solutions (<2 mg mL^−1^) showed low scattering intensities and did not form noodles.

**Fig. 1:**
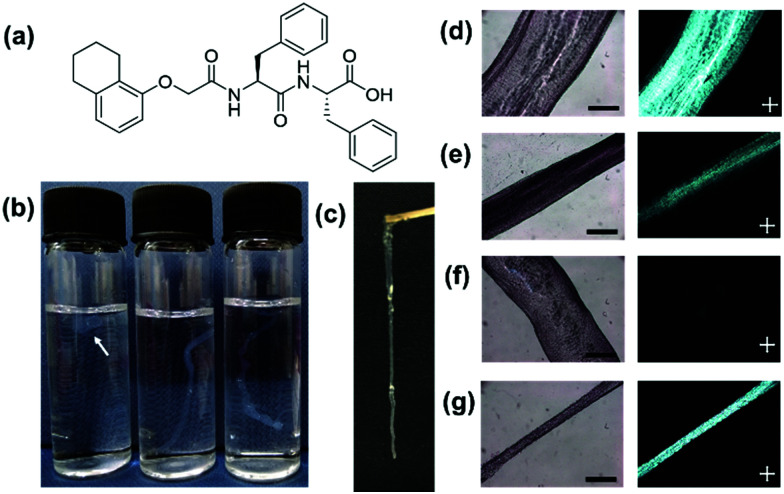
(a) Chemical structure of 1ThNapFF; (b) photograph of gels formed at different injection speeds for the 1 mL pipette. Left vial is a slow injection (with an arrow to guide the eye), the middle vial is an intermediate injection and the right vial is a fast injection; (c) a photograph of a gel filament lifted out of trigger medium; normal and cross polarised light microscope images for (d) 1 mL pipette static; (e) 1 mL pipette dragged; (f) 200 μL pipette static and (g) 200 μL pipette dragged. Scale bars represents 500 μm and the white crosses represent the polariser directions.

A concentration of 10 mg mL^−1^ was selected for most of the experiments. The noodles were sufficiently mechanically robust to be lifted out of the trigger medium and remain intact (Fig. 1c). The parameters that control filament formation were investigated in detail. In previous literature, details of the exact pipettes used to prepare optimal filaments are often not detailed. To investigate the effect of using different pipettes (and therefore changing the pipette tip diameter from which the solution is dispensed), 2–20 μL, 20–200 μL and 100–1000 μL (1 mL) pipettes were tested (Section S2.3, ESI†).

At first, the speed of injection from the pipette was tested (Fig. 1b) in 7 mL vials by varying the rate of depressing the pipette plunger by hand. A faster rate of pressing the plunger, the faster the flow rate of the dipeptide solution leaving the pipette tip. The test was could not be accurately controlled or quantified because it was performed by hand but it gave a valuable insight into the effect of injection speed. It was found that with a slow injection the pipette tip would block or a ‘ball’ of gel would form. For the 2–20 μL pipette, this was not seen and at very slow injection rates noodles were formed. When injected too quickly, turbulence created from the rapid injection resulted in inhomogeneous noodles. With intermediate injection speeds all three pipettes could form continuous, uniform filaments. For filament formation, the 1ThNapFF solution was dispensed into a 100 mL bath of 50 mM CaCl_2_. The resulting noodles formed from intermediate injection speeds were imaged on an optical microscope under both normal and cross polarised light (Fig. 1d–g and Section S2.3, ESI†). Four noodles were made using each pipette and imaged, resulting in >20 representative microscope images. ImageJ was used to measure filament diameter in each microscope image and the result plotted as a histogram (Fig. S20 and Section S2.3, ESI†). The results show a distribution of filament diameters for the 20–200 μL and 1 mL pipette. The 2–20 μL pipette has a relatively narrow distribution centred on 0.45–0.50 mm. Most filaments made were wider than the pipette tip used, which may be the die-swell phenomena.^22,23^ Using cross polarised optical microscopy (CPOM), aligned structures exhibit birefringence and appear bright in an otherwise dark image. Uniformly birefringent sections of filament were seen for the 20–200 μL and 1 mL pipettes in some places (Fig. 1d, f and Fig. S21, Section S2.3, ESI†) but not throughout the structure. The alignment was not achieved consistently and predictably.

We hypothesised that stretching the noodles as they form may help induce alignment. Therefore, noodles were made by a dragging process. This involves dragging the pipette tip through a pool of trigger medium whilst dispensing pre-gel solution. A similar technique (but not with the specific purpose of achieving stretching and alignment) has been reported previously in the literature, where a video can be seen in the ESI of that publication.^11^ The speed at which the pipette is dragged will influence the filament that forms. The dragging technique showed a range of diameters in the same filament and could not be performed reproducibly. With a relatively fast drag, using both the 20–200 μL and 1 mL pipettes a reduction in filament diameter occurred in places. The thin filament regions showed uniform birefringence (Fig. 1e and g). The results suggest that an extensional process as the filaments form can effectively align the nanostructure. Extensional stresses have been shown to be important for the formation of synthetic spider silk, polymer crystallisation and the alignment of anisotropic particles.^24–26^ This dragging technique was unsuitable for the 2–20 μL pipette as the filament was simply pulled along with the pipette. Dispensing by hand when using the pipettes was easy but lacked reproducibility and consistency. Aligned regions were formed both under static and dragged conditions but this was difficult to control and resulted in limited lengths of aligned filament.

To reproducibly control noodle formation, a syringe pump was employed (Section S2.4, ESI†). Briefly, a syringe containing gelator solution was attached *via* tubing to a flat-headed needle (413 um inner diameter) and connected to a syringe pump. With this setup the effect of flow rate, 1ThNapFF concentration, and 1ThNapFF solution pH were investigated. For example, the flow rate can be used to control the filament diameter, where thicker filaments are formed at higher flow rates (detailed data can be found in Section S2.4, ESI†). As with the pipettes, excessively slow (<5 mL hr^−1^) and excessively fast flow rates (>200 mL hr^−1^) did not form uniform noodles. The results show that filament diameter can be controlled reproducibly and changes depending on the parameters used. For any one set of conditions, there is a size distribution in noodle diameter. With this setup, multi-meter long continuous noodles can be easily made (Fig. 2c with the full image in Fig. S30, ESI†) and is limited only by the size of the syringe and the bath into which they are made. As with the pipettes, noodles made by static injection into trigger medium with the syringe pump did not consistently show alignment in CPOM images. Using pipettes, extensional deformation (stretching) the noodles as they formed induced alignment. To do this reproducibly, a spinning technique was developed (Fig. 2a). This used a bath of trigger medium rotating at 100 rpm on a spin coater, into which the pre-gel solution was injected. The spinning procedure did not destroy the structures and instead resulted in a long single continuous noodle (Fig. 2b). The spun noodles had diameter distributions centred on 0.1 mm (Fig. 2d and Section S2.4, ESI†), a quarter of the inner diameter of the needle. Based on a localised rotation speed around the dispensing needle of approximately 2136 cm min^−1^ and a 10 s injection time, the bundle of continuous noodle would be approximately 3.5 m long if unravelled. The spun filaments exhibited strong birefringence in CPOM measurements (Fig. 2e and f), indicating that the spun noodles have aligned nanostructures. By spinning, gel noodles with a narrow size distribution and good alignment were reproducibly formed.

**Fig. 2:**
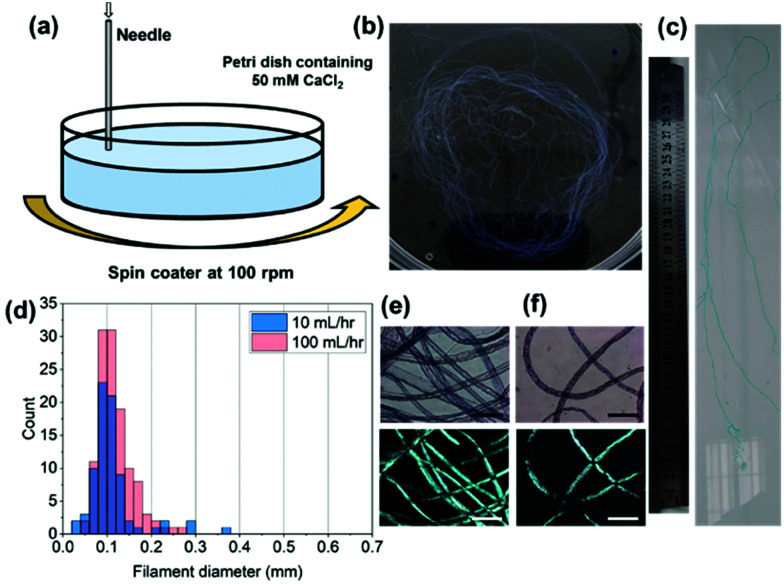
(a) Cartoon of the spinning technique used to form aligned filaments; (b) a photograph of the spun gel noodles in a 9 cm petri dish. (c) Cropped photograph of part of a multi-meter filament with a 30 cm ruler for scale, see full image in Fig. S30 of ESI.† (d) Histogram of filament noodles for 10 and 100 mL hr^−1^; normal and cross polarised light microscope images for spun noodles at (e) 100 mL hr^−1^ and (f) 10 mL hr^−1^. The scale bars in (e and f) represent 0.6 mm.

To the best of our knowledge, no previous work has studied the mechanical properties of LMWG gel noodles although the tensile strength of self-assembled nanotube yarns that are drawn directly from solution has been previously studied.^27^ Some LMWG noodles have been shown to be sufficiently mechanically robust to tie into a knot,^11^ and the ones shown here are self-supporting if lifted out of the solution (Fig. 1c). Using conventional techniques (such as oscillatory rheology), the mechanical properties of the gel filaments cannot be measured due to the small size and 1D shape. Nanoindentation is a technique that can be used to measure the mechanical properties of a localised area (on the μm scale) of a material.^28^ The nanoindenter tip is scanned across the gel surface and >20 individual measurements performed. The force–indentation curve for each measurement is fitted to give a Young's modulus and a median value is calculated for each gel. Using nanoindentation, the Young's moduli of gel filaments and bulk gels (Fig. 3a–c and Section S2.5, ESI†) were measured. The data are presented in violin plots, which show the distribution of the data, with the white dots representing the median. The results (Fig. 3d) show that the Young's moduli for gel filaments at two different flow rates are similar at around 5–20 kPa.

**Fig. 3:**
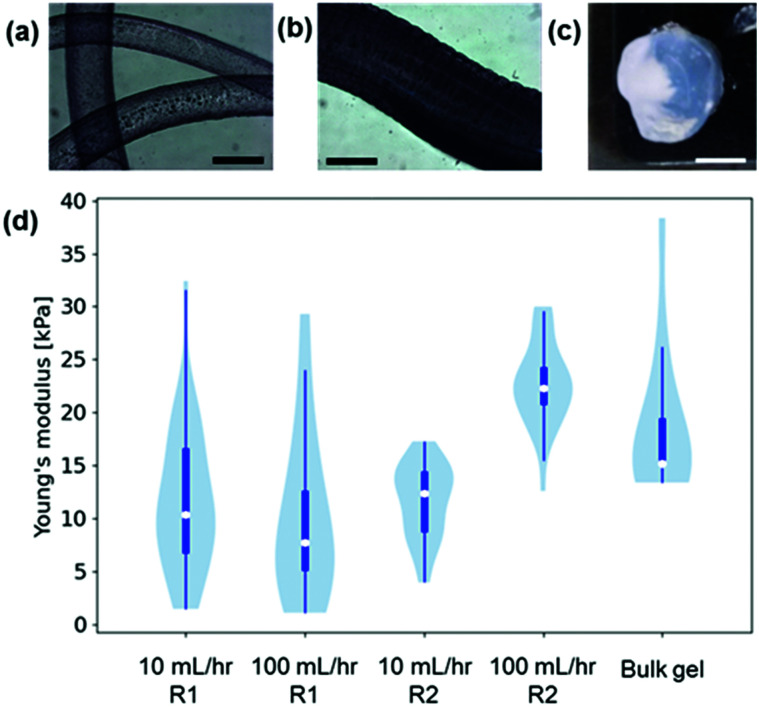
Optical microscope images of gel noodles formed at (a) 10 mL hr^−1^ and (b) 100 mL hr^−1^; (c) a bulk CaCl_2_ gel and (d) violin plots of the Young's moduli of the gel noodles and the bulk gel. For (a and b) the scale bars represent 0.5 mm and for (c) the scale bar represents 7.5 mm.

The Young's moduli are similar to those of the homogeneous bulk gel prepared with CaCl_2_, which shows that the process of noodle formation does not significantly change the mechanical strength of the gels. With the nanoindentation setup available, the spun noodles were too small to be accurately measured.

Finally, with two syringe pumps, a concentric flow setup was created (Section S2.6, ESI†), inspired by a microfluidic setup that was previously used to align V_2_O_5_ nanorods.^29^ Using the trigger medium as the sheath flow and driving the sheath flow by hand, noodles very similar to those formed with the spinning technique can be formed. With this setup, if both flows are loaded with gelator solutions, a noodle-in-noodle morphology can be accessed (Fig. 4).

**Fig. 4:**
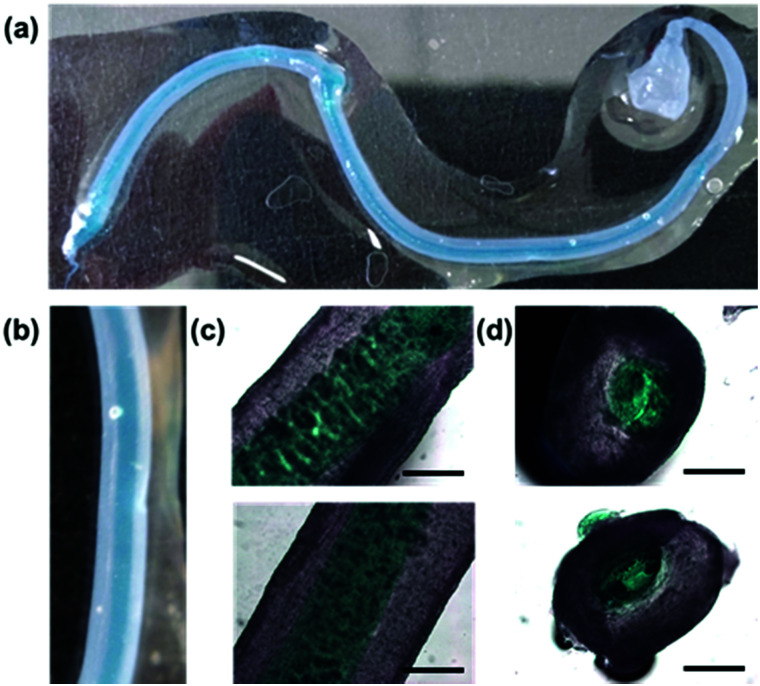
A filament-in-filament morphology formed using a concentric flow setup with sheath of 10 mg mL^−1^ and an inner of 5 mg mL^−1^ (Nile Blue A stained). Photographs of (a) the whole filament and (b) a close-up taken with a macro lens. Optical microscope images of (c) the filaments side-on and (d) cut cross-sections of the filament-in-filaments. The scale bars represent 0.6 mm.

The inner pre-gel solution was stained with Nile Blue A for visualisation. The diameter of the inner noodle as well as the whole noodle can be controlled by varying the two flow rates (Section S2.6.2, ESI†). Cross-sections of the noodle-in-noodle were cut using a scalpel and imaged (Fig. 4d). The inner and outer filament regions were studied with nanoindentation (Fig. S39 and Section S2.5, ESI†). In this experiment, the two components showed similar Young's moduli but conceivably, a noodle-in-noodle morphology with two very different Young's moduli could be formed.

In conclusion, we have studied the parameters that control low molecular weight gel noodle formation. A minimum concentration of 1ThNapFF was required to form gel noodles, which is related to requiring a certain underpinning self-assembled structure and solution viscosity. By using a syringe pump, we can align the nanostructure within the noodles reproducibly using an extensional deformation. A spinning technique was developed to reproducibly form continuous multi-meter long aligned noodles. The noodles are sufficiently mechanically robust to be lifted out of solution. The mechanical properties of the noodles were measured using nanoindentation and show similar Young's moduli to a bulk gel. With a concentric flow set-up, a noodle-in-noodle morphology can be accessed and controlled.

DM thanks the Leverhulme Trust for funding (RPG-2018-013). This work was funded by a grant from the UK Regenerative Medicine Platform. MC acknowledges MRC for funding (MR/S005412/1). This work benefited from the use of the SasView application, originally developed under NSF award DMR-0520547. SasView contains code developed with funding from the European Union's Horizon 2020 research and innovation programme under the SINE2020 project, grant agreement No 654000.

## Conflicts of interest

There are no conflicts to declare.

## Supplementary Material

CC-057-D1CC03378F-s001
