# Supplementary material for: Controlling the formation and alignment of low molecular weight gel ‘noodles’
Source: Chem Commun (Camb). 2021 Aug 4;57(70):8782–5. doi: 10.1039/d1cc03378f (PMC8447933; doi:10.1039/d1cc03378f)
Supplement: CC-057-D1CC03378F-s001 [file CC-057-D1CC03378F-s001.pdf]

Electronic Supplementary Material (ESI) for ChemComm.
This journal is © The Royal Society of Chemistry 2021

Controlling the formation and alignment of low molecular weight gel 'noodles'

Daniel McDowall,[a] Matthew Walker,[b] Massimo Vassalli,[b] Marco Cantini,[b] Nikul Khunti,[c] Charlotte Jennifer Chante Edwards-Gayle,[c] Nathan Cowieson[c] and Dave J. Adams[a]

Supporting Information

Author contributions

Conceptualization (DJA, DM), Data curation (DM, MV, MW, NK, NC, CJCEG), Formal analysis (DM, MW, MV), Funding acquisition (DJA, MV, MC), Investigation (DM, MW, MV, NK, NC, CJCEG), Methodology (DM, DJA, MW, MV, NC, NK, CJCEG), Project administration (DJA, DM), Resources (DJA, DM, MW, MV) Software (MV, MW, MC), Supervision (DJA), Validation (DM), Visualisation (DM, MW, MV), Writing – original draft (DM, DJA), Writing – review and editing (all).

1 Materials & Methods

1.1 Materials

The synthesis and characterisation of 1ThNapFF has been reported previously.[1] $CaCl_2$ was obtained from Alfa Aesar and used with no further purification. Nile blue A was obtained from Sigma Aldrich and a solution in deionised water at a concentration of 8000 ppm was prepared from this.

1.2 Solution preparation

Aqueous solutions of 1ThNapFF were prepared as follows. 1ThNapFF was weighed into a 40 mL plastic Falcon tube followed by the addition of deionised water and then 2 equivalents of 2M NaOH in deionised water. The amounts used varied depending on the volume and concentration required. Solutions were typically prepared on a 10 mL scale. The solutions were stirred overnight and then checked for undissolved solids the following morning and stirred further if required. Once dissolved, the solution pH was checked. The solution pH was adjusted to pH 11.3 ± 0.1 as required with 2M HCl and/or 2M NaOH. Generally, the initial pH >12 and was then lowered to pH 11.3. Care was taken to ensure homogeneity of the sample when pH adjusting. The viscous nature of 1ThNapFF solutions meant that insufficient mixing could result in localised pH differences. Once pH adjusted the solution was ready to use.

To stain the 1ThNapFF solutions blue for enhanced filament visibility when required, a calculated volume of 8000 ppm Nile blue A stock solution was added to achieve a final concentration of 80 ppm Nile blue A in the gelator solution.

1.2.1 Heat/cool cycle

Some 1ThNapFF solutions were subjected to a heat/cool cycle. 10 mL 1ThNapFF solutions were pH adjusted to the required value, then transferred to 14 mL glass vials. The glass vial was placed in a pre-heated and temperature equilibrated oven at the desired temperature (either 60 or 80±2°C). The solutions were kept in the oven for 1 hour, removed and allowed to cool at room temperature for 3 hours.

For forming gel filaments with heat/cooled solutions, the hot gelator solutions were removed from the oven and immediately loaded into a plastic syringe to be used on the syringe pump. The syringe tip was sealed with Parafilm and the solution filled syringe allowed to cool for 3 hours.

1.3 Bulk gel preparation

Bulk gels were prepared for nanoindentation from 10 mg/mL 1ThNapFF solutions at pH 11.3. 2 mL of pre-gel solution was pipetted into an upside down plastic syringe with the top cut off, in a fashion as

had previously reported.[2] This method is favourable because it means the gels can be easily removed once formed and taken for study using nanoindentation. Two equivalents of Ca^{2+} ions (in the form of an 8.9 μL drop of 200 mg/mL $CaCl_2$ in deionised water) was added near the centre of the pre-gel solution. The open top of the syringe was covered with Parafilm. Localised gelation where the $CaCl_2$ droplet was placed occurred instantly. For gelation to occur throughout the sample, the Ca^{2+} diffuses through the solution. For this particular system the diffusion was an extremely slow process and even when left for many months a completely homogeneous gel is not formed. For the nanoindentation experiments two bulk gels were studied. An inhomogeneous bulk gel, for which diffusion had been left for 7 days, and a homogeneous gel that was left for 6 months. Although it is noted that the homogeneous gel was still not entirely homogeneous by eye. For the 6-month diffusing gel, this was instead prepared on a 2 mL scale but in a 7 mL Sterilin vial to allow for an effective seal and prevent drying of the gel. The inverted syringe technique cannot be sealed effectively, especially for many months. Instead, the gels dry out.

1.4 Viscosity measurements

Viscosity measurements were made using an Anton Paar 301 rotational rheometer with a 50 mm cone and plate geometry. All measurements were performed at 25°C and a shear rate range of 1-1000 s^{-1}. To minimise the shear that the 1ThNapFF solutions are subjected to prior to the experiment, they were loaded onto the rheometer by pouring instead of using a pipette. Using a pipette can shear-align the sample and change the measured viscosity.

1.5 Pipette filament formation

All gel filaments were analysed on the day they were made unless stated otherwise.

1.5.1 Pipettes used

An Eppendorf 100-1000 μL (1 mL) pipette, SLS Lab Basics 20-200 μL pipette and SLS Lab Basics 2-20 μL pipette were used.

2-20 μL pipette tips: Fisherbrand SureOne aerosol barrier pipette tips (product code 11977714)

20-200 μL pipette tips: Eppendorf epTIPS standard pipettor tips (product code 11920537)

100-1000 μL pipette tips: Fisherbrand, polypropylene blue pipette tip (product code 10787524)

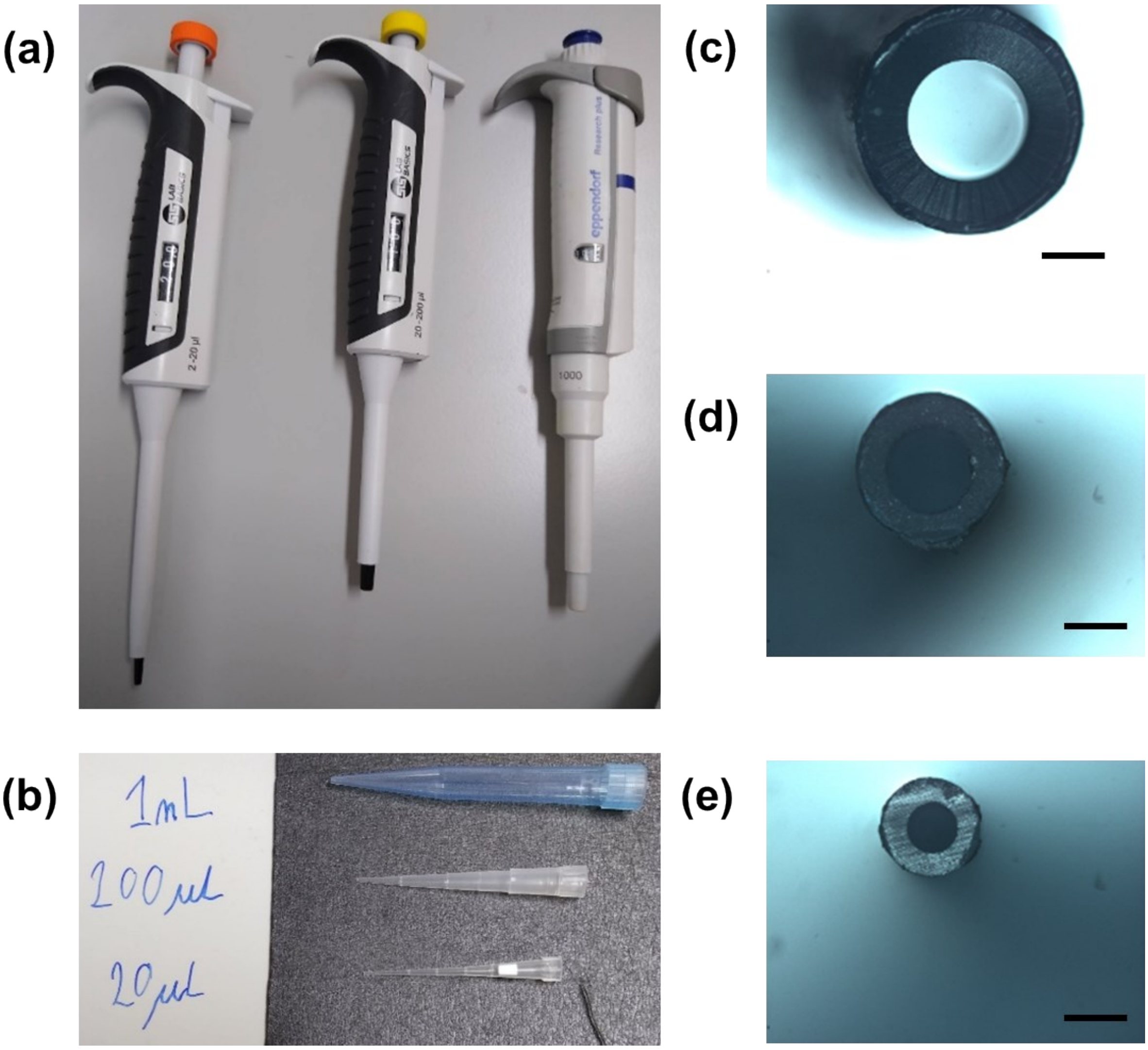

Figure S1. (a) Pipettes used. From right to left, 100-1000 µL (1 mL), 20-200 µL and 2-20 µL; (b) photograph of the pipette tips; Optical microscope images of pipette tips used (c) 100-1000 µL (1 mL); (d) 20-200 µL and (e) 2-20 µL. Images at 5X magnification, under normal light, in reflectance mode. Scale bars represent 0.4 mm.

1.5.2 Static filament formation

With 1ThNapFF solution loaded into a pipette, gel filaments were formed by submerging the pipette tip vertically into a 100 mL bath of 50 mM $CaCl_2$ and dispensing the pre-gel solution. This resulted in the formation of a continuous filament of gel until all the pre-gel solution was dispensed or the injection stopped. For the 2-20 µL pipette 20 µL of 1ThNapFF solution was used. For both the 1 mL and 20-200 µL pipettes, 100 µL 1ThNapFF solution was used. A process of trial and error to achieve an intermediate flow rate that result in uniform filaments was required for each pipette. The Ca^{2+} ions were in approximately a 1000 times excess relative to the 1ThNapFF molecules.

1.5.3 Dragging pipette

Filaments were formed by dragging the pipette tip along a microscope slide with a pool of 50 mM $CaCl_2$ atop whilst dispensing pre-gel solution. The general technique was inspired by a video in the supporting information of work by Zhang *et al.*,[3] but it was seemingly not performed with the intention of inducing alignment in that work. Here, a faster dragging than shown in that video was performed.

1.6 Syringe pump filament formation

All gel filaments were analysed on the day that they were made unless stated otherwise.

1.6.1 Syringe pump

An Alaris Carefusion syringe pump was used to control the flow rate of the 1ThNapFF pre-gel solutions. A 10 mL syringe was attached via a Luer lock fittings to 20 cm rubber tubing with a flat-headed needle (413 µm inner diameter) at the end. To load the syringe pump, the syringe was filled directly with gelator solution. The tubing was attached to the syringe and the pre-gel solution pumped manually until the tubing was full with pre-gel solution. The needle was then attached to the end of the tubing and the syringe loaded into the syringe pump. The rubber tubing and needle enabled movement of the needle to position it into the trigger medium. Next, the syringe pump was started until the pre-gel was coming out. During this process, care was taken to avoid the formation of bubbles within the tubing.

1.6.2 Static filament formation

To make gel filaments, a cylindrical Pyrex dish with a microscope slide at the bottom was filled with 100 mL 50 mM $CaCl_2$ trigger solution. A flow rate was selected and the infusion started. The first drops of pre-gel solution was dabbed onto tissue paper and then the needle moved and positioned vertically downwards into the trigger medium ~5 mm below the surface. The infusion was carried out for 15 s. To finish the needle was slowly moved to the edge of the dish and then dragged up the side to break the connection to the rest of the structure. As the filaments were generally one continuous structure, simply pulling the needle out may pull the whole structure and alter/damage it. After removing the needle, the syringe pump was stopped. Once formed the filament gel structures became more opaque over the course of a minute. The excess trigger solution was carefully removed manually from the dish with a 50 mL syringe. When the level of trigger solution was reduced to around 20 mL the floating gel structures were carefully positioned above the microscope slide. The remaining trigger solution was then slowly removed so that the gel structure “landed” on the microscope slide. The microscope slide was then taken for microscope imaging. The filaments were never left to dry out and a few drops of trigger medium added atop the microscope slide to keep them hydrated.

The 1ThNapFF solutions, in ambient room lighting, were colourless and the filaments were difficult to see for the first few seconds but became slightly opaque as gelation occurred. A flashlight positioned at

the side shining into the dish allowed for viewing of the filaments as they form. The syringe pump was always started before placing the needle into trigger solution. If the infusion was not started when the needle was placed in trigger solution the trigger could travel up the needle, forming a gel and blockage inside the needle. As the infusion was started before immersion in the trigger, there was often a droplet of gelator solution on the needle when it was placed into the trigger. When immersed into the trigger this droplet gelled resulting in a ball shaped gel structure on the tip. A small and fast shake of the needle tip in solution dislodged this allowing for the fluid to elute into solution and form filament structures. This shake did not affect the formation of the subsequent filament structures.

1.6.3 Spinning aligned filaments

Aligned gel filament structures were formed through a spinning technique. 20 mL trigger solution was pipetted into the bottom half of a 10 cm Petri dish. Dots were drawn on the underside of the Petri dish 1 cm in from the edge. This was secured with Blu-tack on a spin coater. It was important to position the Petri dish as close to the centre of rotation as possible to prevent the non-uniform flow of trigger medium. A speed of 100 rpm was used, resulting in a localised “velocity” of trigger medium around the needle of approximately 2136 cm/min.

The spin coater (from Ossila) was started and after a few seconds the solution in the Petri dish would reach an equilibrium point, forming a uniform ring of fluid around the edges of the dish. The infusion of the pre-gel solution from the syringe pump was started, and then placed vertically into the rotating trigger solution above the marked points 1 cm in from the edge of the dish. Any gel droplet present on the end of the needle was removed by the flow of fluid around the needle tip. The injection into the Petri dish was performed for 10 seconds. The needle was removed as closely to the top of the Petri dish as possible. Pulling the needle directly up and away may result in thin filaments of gel that fall into the solution, leading to unrepresentative results when imaged and measured. Following removal of the needle the spin coater was stopped. The bundle of gel filament structures could then be carefully manipulated in the trigger solution. A microscope slide was carefully placed in the Petri dish avoiding touching the gel structures. The gel filament structures were then carefully positioned over the microscope slide and the trigger slowly removed from the dish leaving the bundle of gel filaments on top of the slide ready to be imaged.

1.6.4 Concentric flow setup

Two concentric flows (an inner and a sheath flow) was created using two syringe pumps by taking the rubber tubing for the sheath flow and piercing it with the inner flow needle (Figure S2 and S3). Through careful alignment and clamping the needle and the sheath tubing can be concentrically aligned. A metal wire can be used to help shape the tubing and facilitate the positing and alignment of the tubing and needle (Figure S3). Care was taken to assure the two flows were concentrically aligned. This was done

with the front camera of a mobile phone and a clip-on mcarolense to help focus on the small object. Pictures were taken to show the good alignment of the two nozzles (Figure S4c and S5c). The exit nozzles of the concentric flows were submerged in a bath of trigger medium by raising the 50 mM $CaCl_2$ bath up to the static nozzles using a mechanical jack (Figure S4a and S5a). As before, the syringe pump flow was started and run until drops were coming out prior to submersion in the trigger medium to prevent blockages. Again, droplet of solution that may be present upon submersion and consequently gelled was removed by a spatula. After this the filament formation would start given sufficient flow rates.

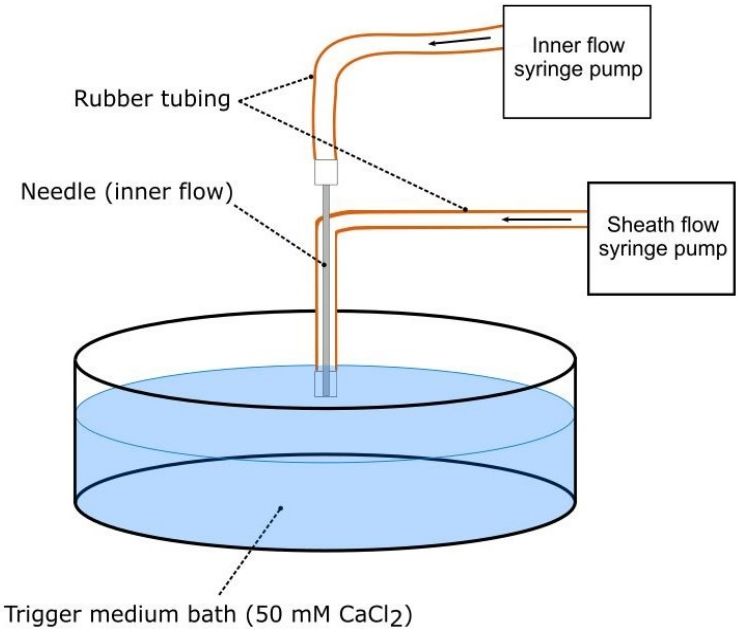

Figure S2. Cartoon schematic of the concentric flow setup.

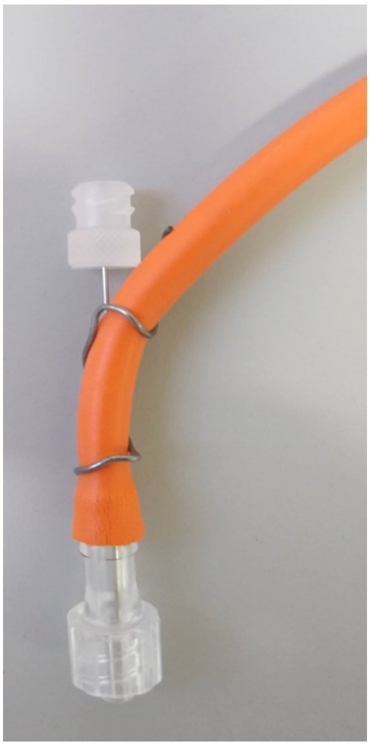

Figure S3. Photograph showing the alignment of a needle within the rubber tubing and Luer-lock nozzle for the sheath flow. Note the metal wire is shaped to manipulate the rubber tubing to aid with this.

1.6.5 <u>Formation of aligned filaments with the concentric flow setup</u>

With the sheath flow as 50 mM $CaCl_2$ and the inner flow as 1ThNapFF solution, the sheath flow can be used to extensionally deform the 1ThNapFF filaments as they form, provided the sheath flow is fast enough. This is discussed more details in the results section of the supporting information.

(a) (b) (c)

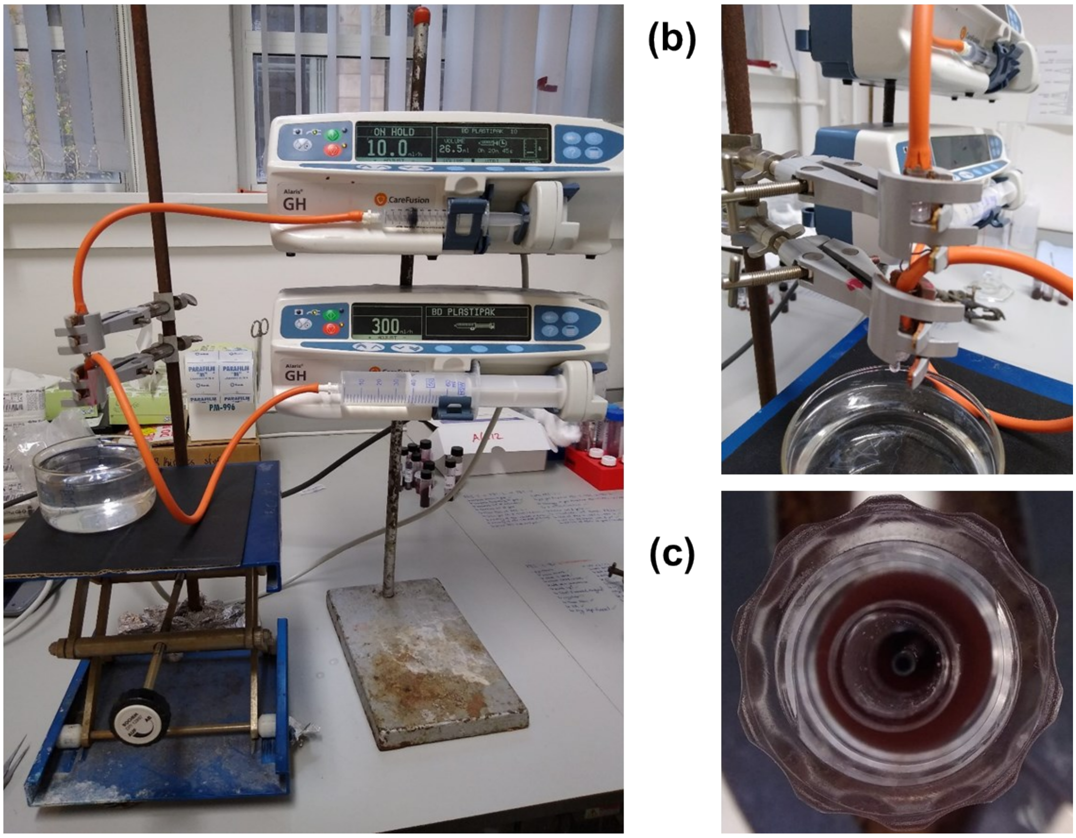

Figure S4. Photographs of the concentric flow setup. (a) Whole setups; (b) close up of tubing and (c) view from underneath showing the concentric alignment of needle and sheath flow. (c) was taken using a clip-on macrolense.

1.6.6 Concentric flow setup: Filament within a filament

Using a similar setup but having both the sheath and inner flows as 1ThNapFF solutions, a "filament-in-filament" could be formed. Here, a 1 mL pipette tip was used for the sheath flow (Figure S5c) as opposed to a Luer-lock connector as above. The pipette tip was cut approximately 1 cm from the exit of the tip. This shortened tip was fitted into the rubber tubing so the exit nozzle from the tubing was the plastic pipette tip. Then the inner flow needle was pierced through the tubing and aligned within the pipette tip from which the sheath flow was dispensed.

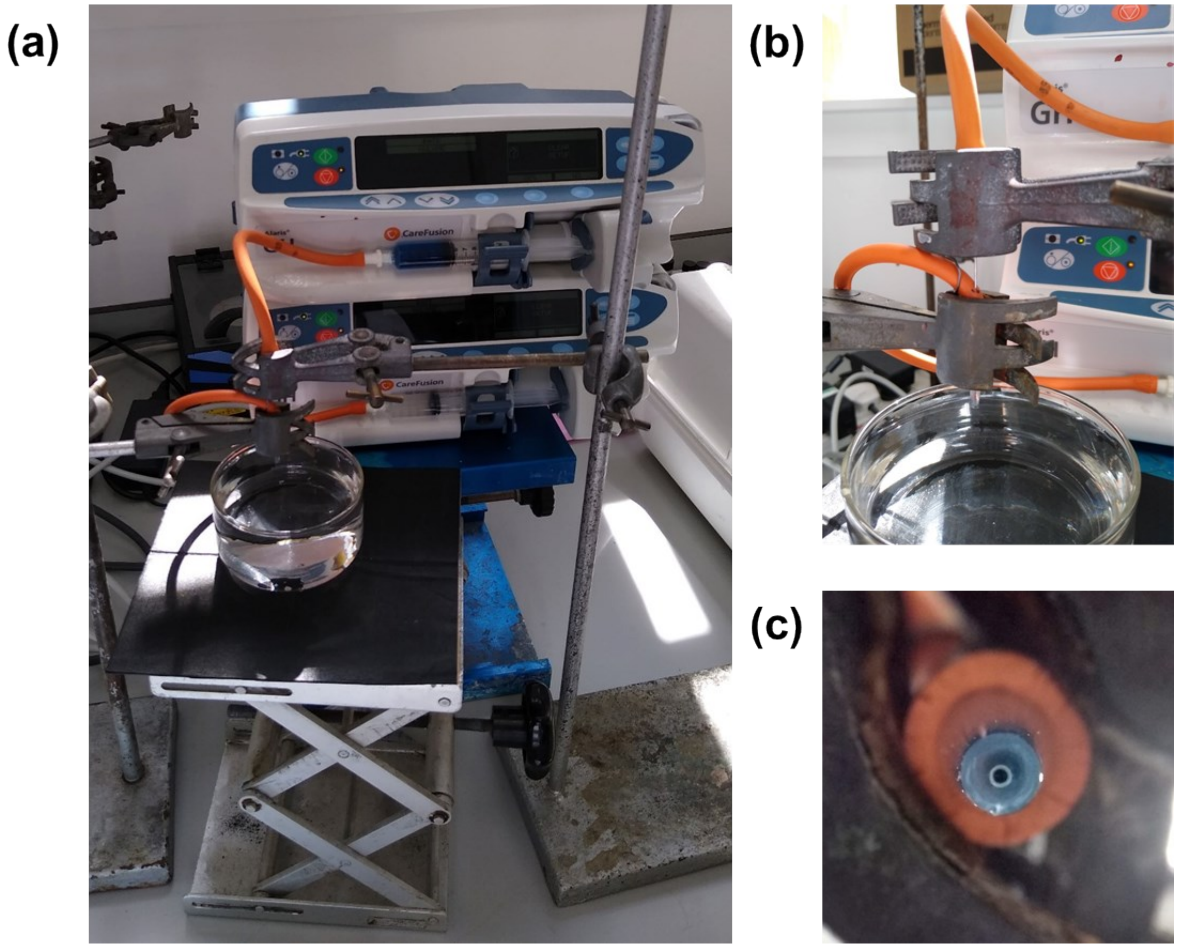

Figure S5. Photographs of the concentric flow setup. (a) Whole setups; (b) close up of tubing and (c) view from underneath showing the concentric alignment of needle and sheath flow.

For this setup an inner flow of 5 mg/mL 1ThNapFF solution and a sheath flow of 10 mg/mL 1ThNapFF solution were selected. The 5 mg/mL 1ThNapFF solution was stained blue with 80 ppm of Nile blue A to distinguish the two layers. Once formation of the filament occurred, the inner flow could be adjusted to vary the thickness of the blue, 5 mg/mL, inner filament within the 10 mg/mL sheath filament.

1.7 Optical microscopy

A Nikon Eclipse LV100 optical microscope with an Infinity 2 camera connected to a computer was used to image the gel filaments. The microscope was used in transmission mode, 5X magnification (LU Plan Fluor lense) and images taken under both normal and cross polarised light. Approximately 20-50 images were taken of each sample. In all CPOM images the direction of polarisers are vertical and horizontal relative to the image. The CPOM light intensity was kept constant by marking the dial positions required for each intensity.

Once the gel structures had been formed and collected on a microscope slide they were imaged. The underside of the slide was dried thoroughly with paper towel and placed on the microscope stage. Droplets of trigger medium was placed on the filament structures to form a pool to keep them hydrated. The gels shrink and lose alignment if not re-hydrated over a 40 minute period (Figure S6). Additionally, this removed any curved water/air boundaries from near the filament structures which resulted in poor images due to the refraction of light (see Figure S6c for this effect visualised).

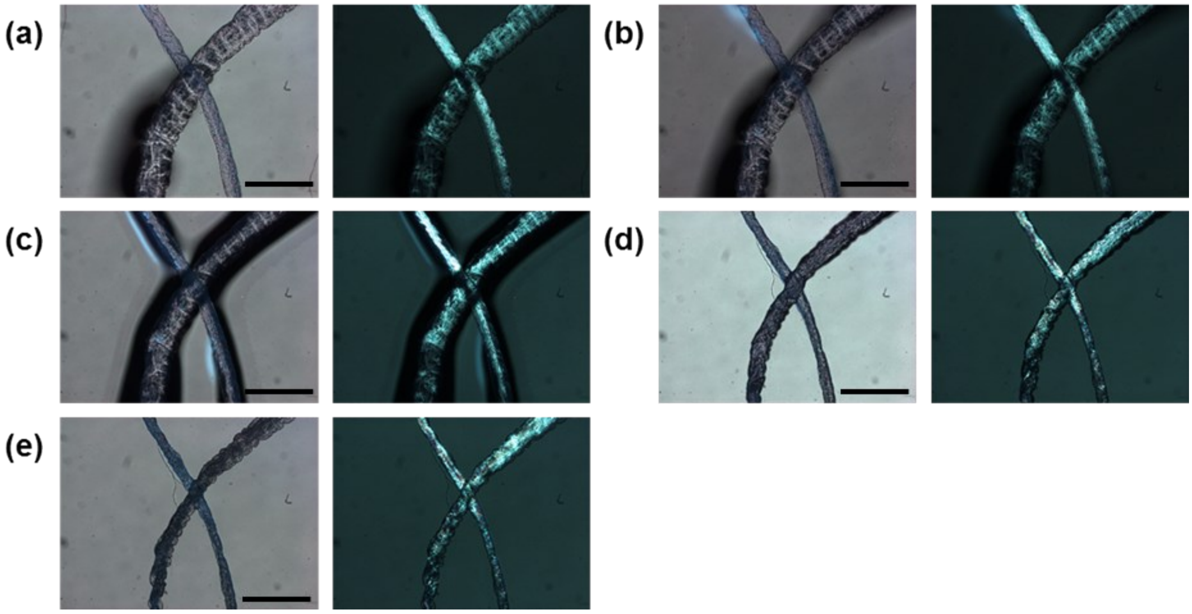

Figure S6. Optical microscope for the drying test showing normal (left) and cross polarised (right) light images at 5X magnification. Images were taken at (a) 0 minutes; (b) 10 minutes; (c) 20 minutes; (d) 30 minutes; (e) 40 minutes. Black scale bars represent 0.6 mm.

1.8 ImageJ analysis

ImageJ (version 1.50e)[4] analysis was used to measure the structure diameters in all of the microscope images. This was done using a 1 mm scale bar supplied with the microscope which is imaged at the same magnification, with which the global scale (pixels/mm) can be set in ImageJ. Out of focus structures were not measured. In each microscope image the diameter of every in focus filament structure was measured at a point approximately in the middle of the structure in view. This allowed for a histogram of filament diameter to be plotted for each sample.

1.9 Nanoindentation

Nanoindentation was used to measure the Young's modulus of the low molecular weight gel filaments and bulk gels. The Young's modulus can vary depending on the experimental setup used.[5] During a nanoindentation experiment, the tip performs a set distance indentation into the sample and the resistance to the deformation is measured. This allows for a force-indentation curve to be created, which is then fitted using a Hertzian model to obtain a Young's modulus. The technique is localised, therefore

the tip is scanned across the surface of the sample and many individual measurements collected. These are then processed and presented all together for each sample. A Chiaro nanoindenter (from Optics11Life) was used for nanoindentation under the indentation control operation mode. Tip radii of 8.5-10 μm, probe stiffness of 0.46-0.51 N/m and a Geo factor range of 1.21-1.25 were used. A maximum displacement of 10,000 nm was used, which includes the whole range including the out of contact region (flat baseline prior to reaching the surface). The indentation was performed at a constant speed of 5 μm/s. The forward segment of the force-distance curves were analysed and filtered for high frequency noise and evaluated with a custom-build software[6] developed using python and available online under open source license.[7] The fitting procedure was applied to the first 2000 nm after the contact point, keeping within 10% of the radius of the spherical indenter tip and 10% the thickness of the sample. A contact point method in the analysis software called 'Goodness of fit' was used.[8] The measurements were performed in 50 mM $CaCl_2$ solutions.

The thin (<0.2 mm), spun gel filaments could not be measured using the specific setup available.

1.10 Small angle X-ray scattering

Small angle X-ray scattering experiments were performed at the B21 beam line at Diamond Light Source, Didcot, UK. The beamline operated at a fixed energy of 13.02 keV, a camera length of 3.7046 m, resulting in a Q range of 0.0026 – 0.34 $Å^{-1}$. The beam flux was $4x10^{12}$ photons per second. The beam is offset to the top right hand corner of the Eiger 4M in-vacuum detector (Dectris). Further details of the SAXS setup can be found in a 2020 publication by the beamline scientists.[9]

The samples were measured in polyimide capillaries. To load the samples into the capillaries, a 1 mL syringe and a 19G needle (inner diameter = 0.686 mm) were used for each solution. For each sample 20 x 1 s frames were collected.

The data was processed in the Dawn Science software (version 2.20.0).[10] As part of the processing procedure, a background of air (an empty capillary) and of the solvent (deionised H_2O in a capillary) were subtracted. The 20 frames were averaged and, for samples that scattered isotropically, a full azimuthal integration performed. Where necessary, flare from capillary mis-alignment was masked out. The azimuthal integration yielded the data as I vs *q* plots. These data were then fitted to structural models in the SasView software (version 5.0.2).[11] Many of the samples exhibited anisotropic scattering, which is consistent with aligned structures within the capillaries.[12] This is attributed to shear-aligning when loading the sample into the capillaries. The anisotropic data cannot be azimuthally integrated as is done for a normal isotropic data set. Instead, bow-tie integrations were performed over the regions of high scattering intensity and low scattering intensity. This results in two separate I vs q plots. The I vs q plots for the high scattering intensity regions were fitted to structural models in SasView. When performing a bow-tie integration over the high intensity (vertical) regions, there are kinks in the data

due to boundaries between the detector modules. This results in anomalous data points which have been manually removed and gaps in the curve. There are some limitations in this approach because the form factor fitting assumes that the self-assembled structures are non-interacting and randomly oriented. For aligned samples the structures are not randomly oriented. While this approach is not ideal and may lead to some small errors in the fitting, it is not significant enough to not fit the data. Indeed, by virtue of the fact that the particles have aligned, this reinforces the application of a structural model consistent with 1D structures. For the fitting X-ray scattering length densities (SLDs) were calculated using the NIST neutron activation and scattering calculator.[13] An SLD of 14.025x10^{-6} Å^{2} was used for 1ThNapFF and a solvent SLD of 9.469x10^{-6} Å^{2} was used.

2 Results

2.1 Rheology

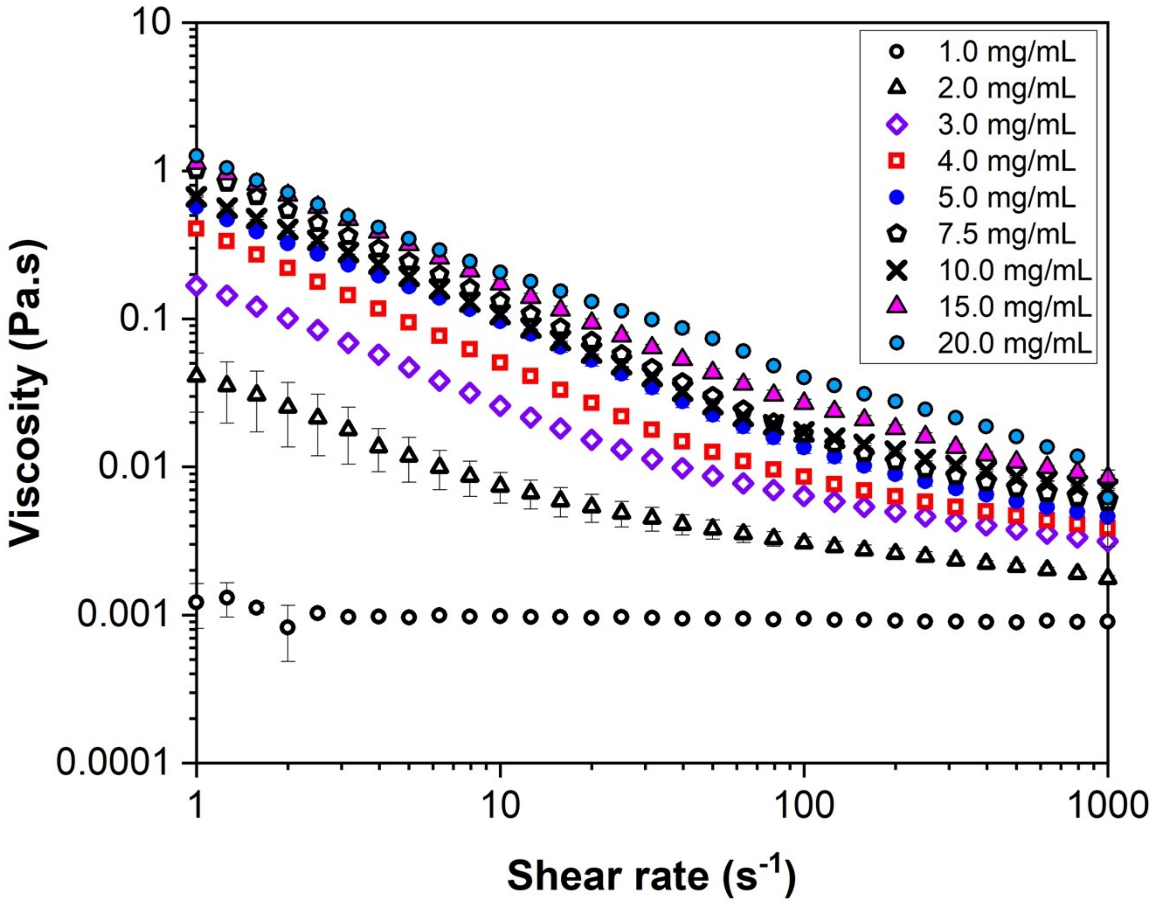

Figure S7. Viscosity of 1ThNapFF solutions at different concentrations at pH 11.3 across a shear rate of 1 to 1000 s^{-1}. Error bars are the standard deviation of triplicate measurements.

The influence of solution pH on the shear viscosity was investigated and showed that across a relatively broad pH range (9.2 – 12.0) the viscosity is broadly unchanged (Figure S8). At pH 8.6 there is a

significant drop in viscosity which is likely attributed to a change in the self-assembled structures present, or a change in the interactions between them.

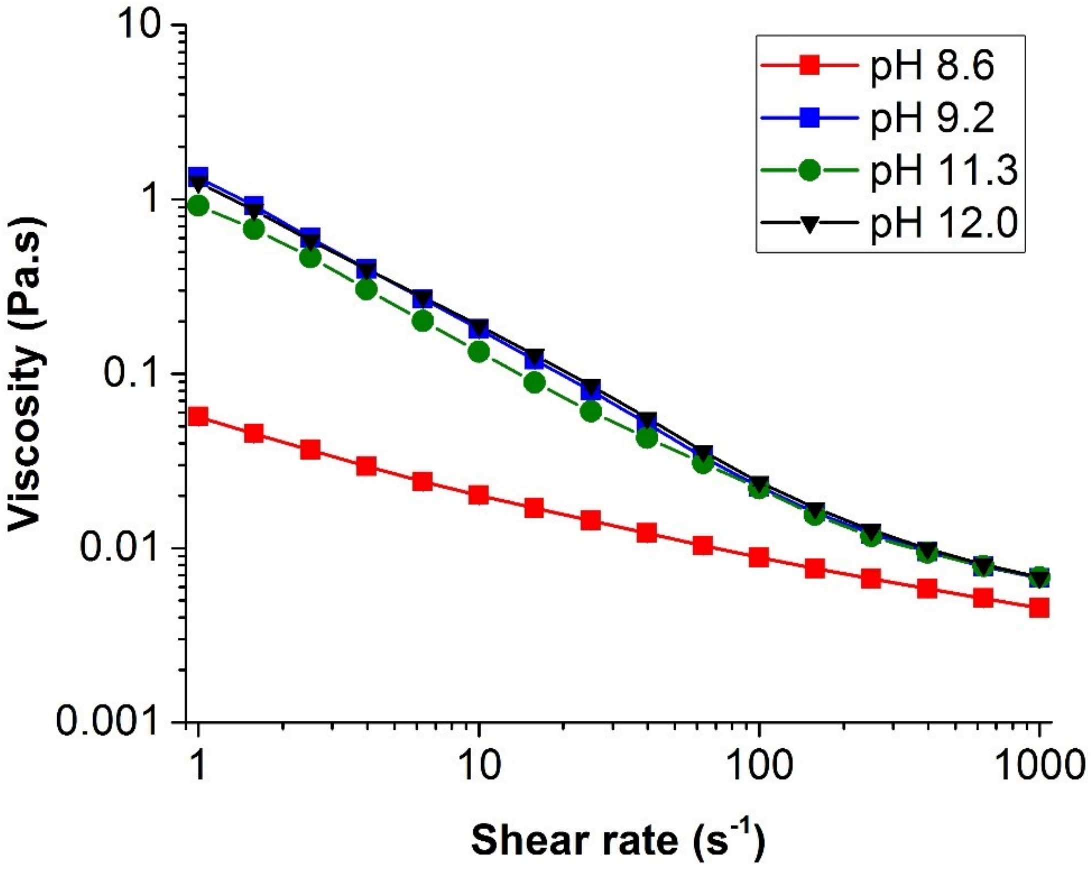

Figure S8. Influence of solution pH of 10 mg/mL 1ThNapFF on viscosity.

The shear viscosity of 10 mg/mL 1ThNapFF solutions at pH 11.3 after heat/cool cycles was investigated and showed that after 60°C the shear viscosity was slightly reduced but after 80°C the viscosity was increased.

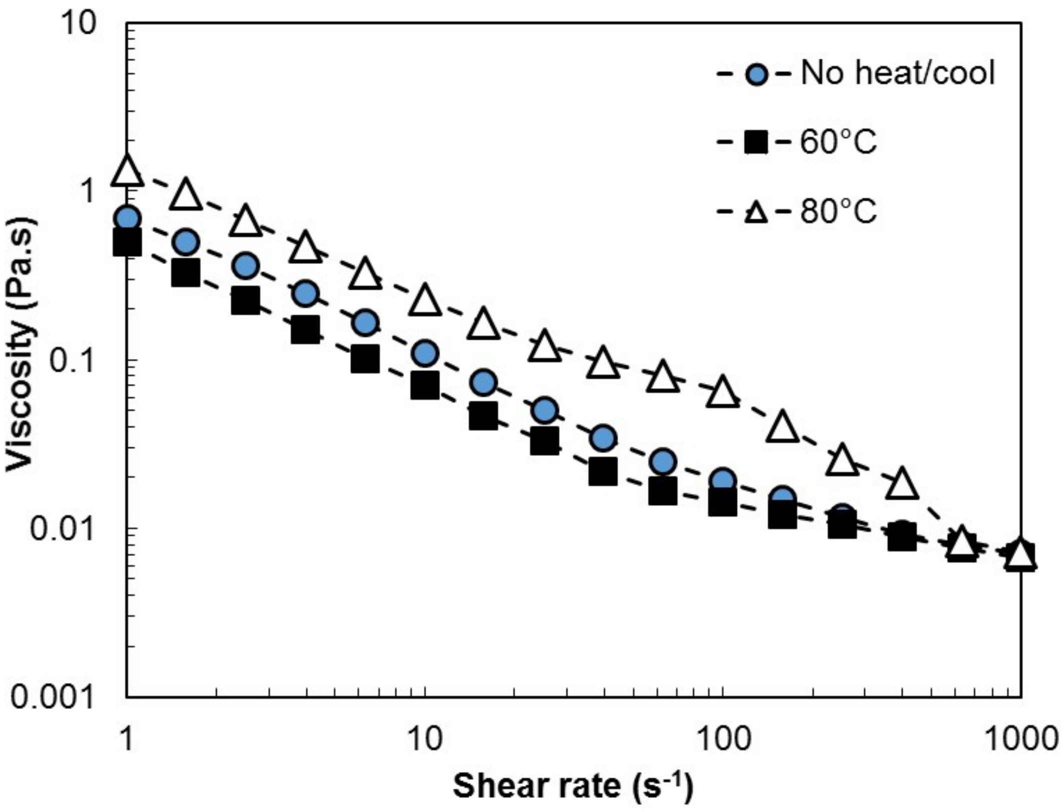

Figure S9. Influence of solution a heat/cool cycle at different temperatures on 1ThNapFF on viscosity.

2.2 Small angle X-ray scattering

All samples at a concentration of >1.5 mg/mL scattered strongly but many show strong anisotropic scattering. The 2D scattering patterns of all data collected are shown in Figure S10 and S11.

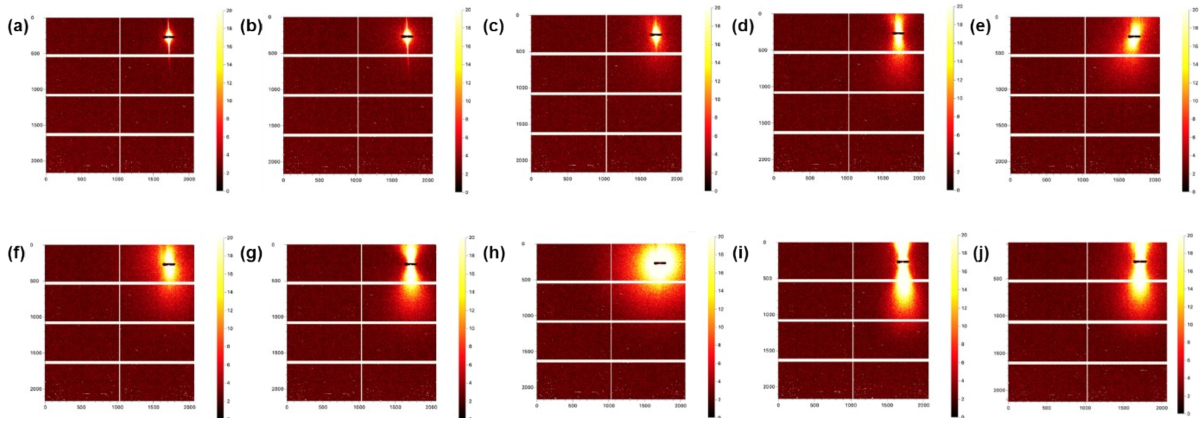

Figure S10. 2D scattering plots of aqueous 1ThNapFF at pH 11.3 at a range of concentrations. (a) 1.0 mg/mL; (b) 1.5 mg/mL; (c) 2.0 mg/mL; (d) 2.5 mg/mL; (e) 3.0 mg/mL; (f) 4.0 mg/mL; (g) 5.0 mg/mL; (h) 7.5 mg/mL; (i) 10 mg/mL; (j) 15 mg/mL.

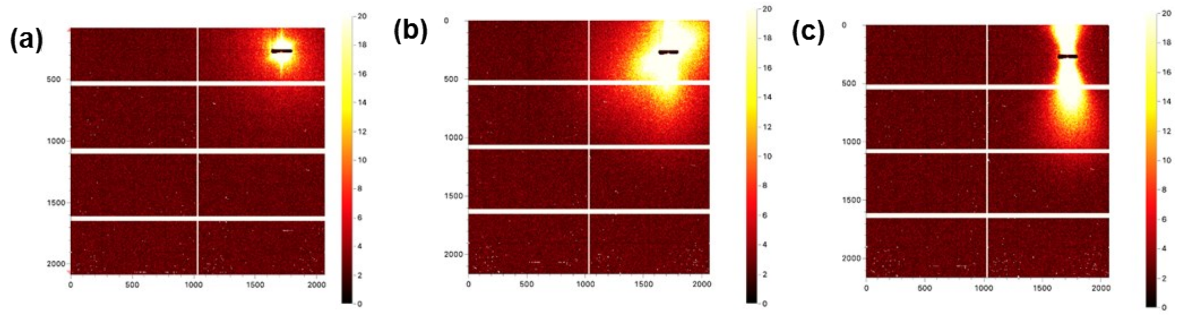

Figure S11. 2D scattering plots of aqueous 1ThNapFF. (a) 2 mg/mL with Nile Blue A (80 ppm); (b) 10 mg/mL at pH 9.2 and (c) 10 mg/mL at pH 12.0.

2.2.1 Fitting data

Data that exhibited isotropic scattering were azimuthally integrated and the resultant plots fitted structural models in SasView as has commonly done before for these type of systems.[14,15] The data for the 1.0 mg/mL and 1.5 mg/mL solutions exhibited low scattering intensity and were not fitted to any structural models. Many of the data show anisotropic scattering patterns in the 2D data. This is attributed to shear-alignment of 1D structures in the 1ThNapFF solution as it is injected into the capillary. As discussed in the experimental, bow-tie integrations were performed. An example of the two I vs q plots obtained from this process is shown in Figure S12. For many of the data a large upturn in intensity at the lowest Q was seen. This has previously been attributed to scattering from the network for self-assembled functionalised dipeptides.[16] For each fit, this low Q region was initially excluded from the fitting and the rest of the data fitted to different cylinder models until a good fit was achieved. For these data typically a cylinder or flexible cylinder model was required to achieve a good fit. This is consistent with the presence of self-assembled worm-like micelles. After this, the values obtained with this flexible cylinder fit were input into a combined flexible cylinder + power law model and the whole Q range fitted. The power law model generally captured this upturn in intensity at low Q.

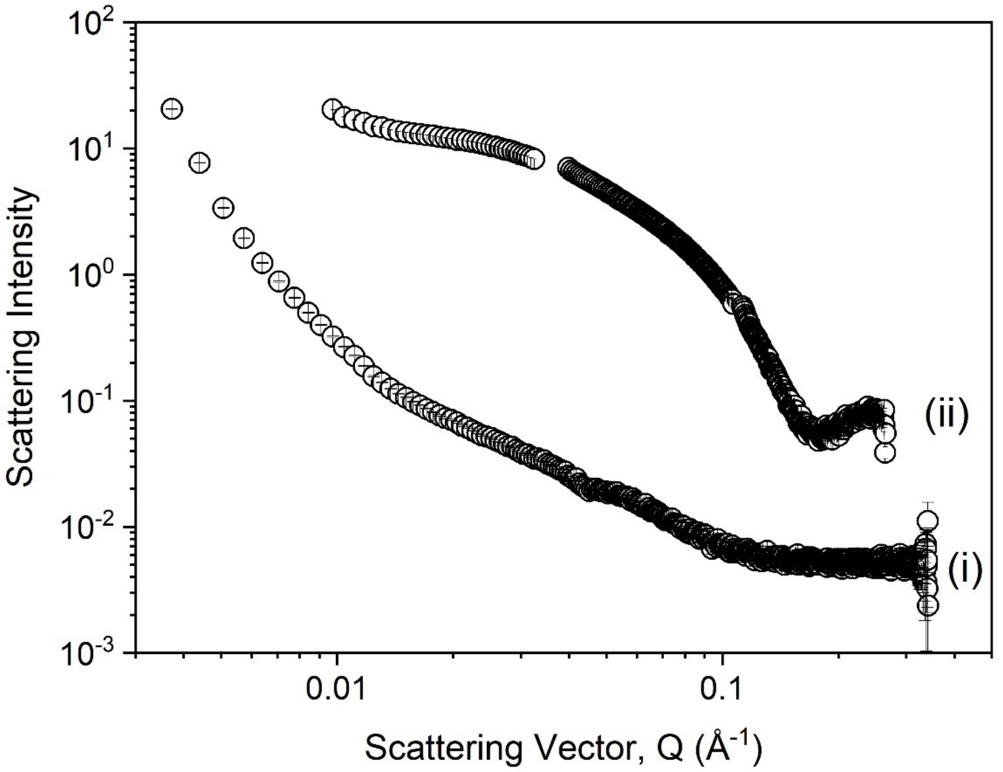

Figure S12. 10 mg/mL pH 11.3 solution bow-tie integration data. (i) the low intensity scattering regions obtained from a horizontal bow-tie integration and (ii) the high intensity scattering regions obtain from a vertical bow-tie integration.

The fitting data for a range of samples in the concentration range of 1 – 15 mg/mL are shown in Figure S13. Importantly, some of these data sets were from isotropic data and others from anisotropic data. Details are given in Table S1.

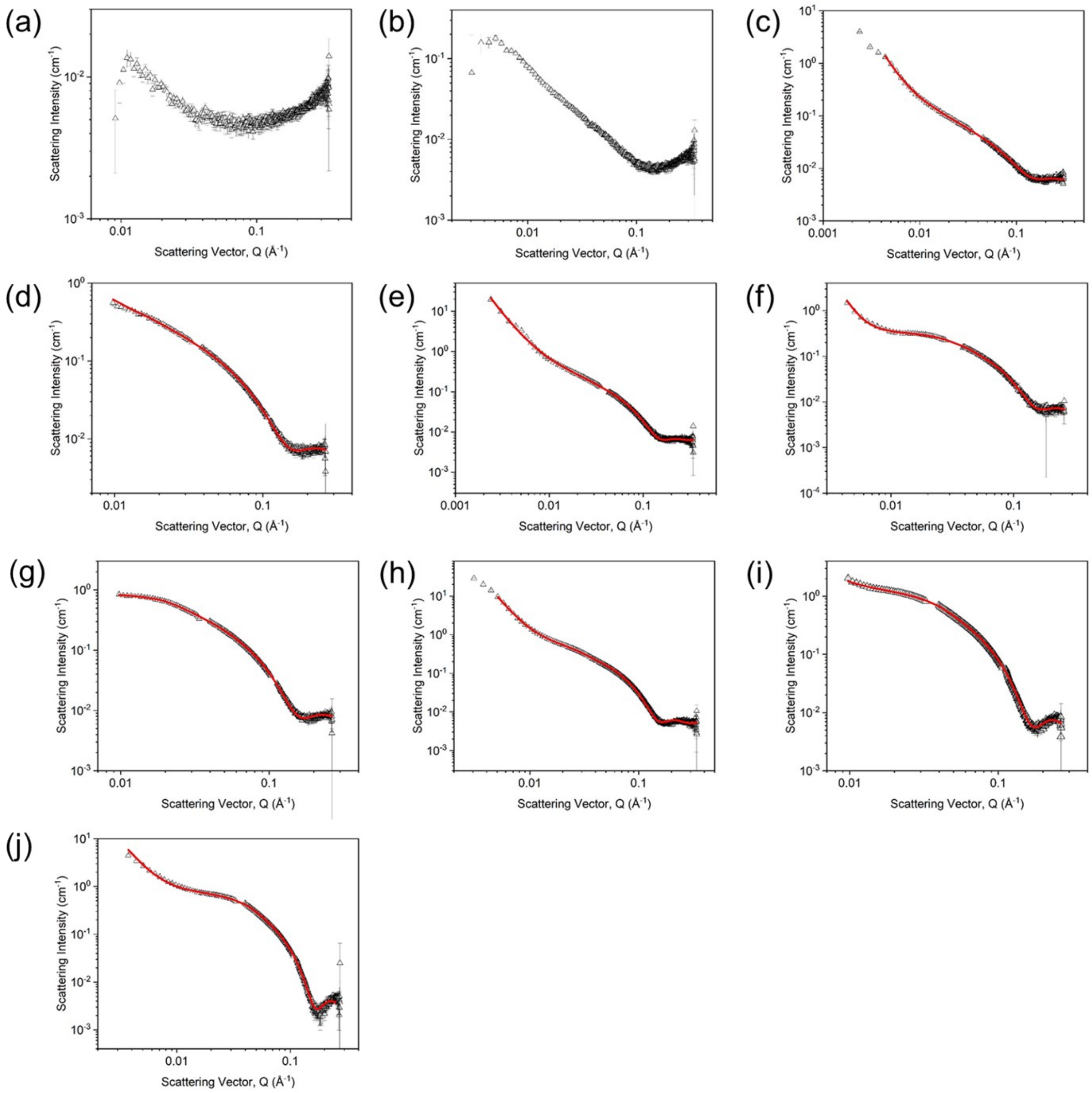

Figure S13. SAXS data (black hollow triangles) and fits (red lines) from the vertical bow-tie integrations of the 1ThNapFF concentration series at pH 11.3. Concentrations of (a) 1.0 mg/mL, (b) 1.5 mg/mL, (c) 2.0 mg/mL, (d) 2.5 mg/mL, (e) 3 mg/mL, (f) 4 mg/mL, (g) 5 mg/mL, (h) 7.5 mg/mL, (i) 10 mg/mL, (j) 15 mg/mL.

Table S1. Processing details and fitting parameters for the concentration series of 1ThNapFF.

Concentration / mg/mL	2.0	2.5	3.0	4.0	5.0	7.5	10.0	15.0
Processing type	Vertical bow-tie integration	Vertical bow-tie integration	Vertical bow-tie integration	Vertical bow-tie integration	Vertical bow-tie integration	Isotropic	Vertical bow-tie integration	Vertical bow-tie integration
Fitting model	Cylinder + power law	Cylinder	Cylinder + power law	Cylinder + power law	Flexible cylinder	Flexible cylinder + power law	Flexible cylinder + power law	Flexible cylinder + power law
Cylinder scale	0.000001	0.000646	0.000493	0.000752	0.001381	0.000964	0.002830	0.001737
Cylinder scale error	0.00000001	0.0000025	0.0000017	0.0000035	0.0000027	0.0000015	0.0000103	0.0000042
Background	0.000035231	0.0070	0.0063	0.0066	0.0075	0.0048	0.0052	0.0027
Background error	0.0000002	0.00005	0.00003	0.00008	0.00005	-	0.00010	0.00005
Radius / Å	21.8	22.4	22.7	22.9	22.2	19.4	21.7	22.1
Radius error / Å	0.14	0.06	0.05	0.07	0.03	0.02	0.03	0.04
Length / Å	798.2	581.1	808.4	133.7	199.9	295.4	149.5	138.7
Length error / Å	59.9	19.9	23.1	0.6	0.7	0.7	0.9	0.9
Kuhn length / Å	-	-	-	-	162.08	73.84	82.43	76.10
Kuhn length error / Å	-	-	-	-	1.34	0.00	0.34	0.54
Power law scale	0.000000000031	-	0.00000007539	0.00000000001	-	0.00000003077	0.00000479480	0.00000026356
Power law scale error	0.0000000000079	-	0.000000003406	0.000000000003	-	0.000000000655	0.000002383000	0.0000000397
Power law	3.51	-	3.21	4.74	-	3.70	2.52	3.00
Power law error	0.04887	-	0.0081	0.0689	-	0.0042	0.11	0.03
Reduced Chi^2	0.33	0.86	5.49	1.90	1.73	25.51	3.85	3.56

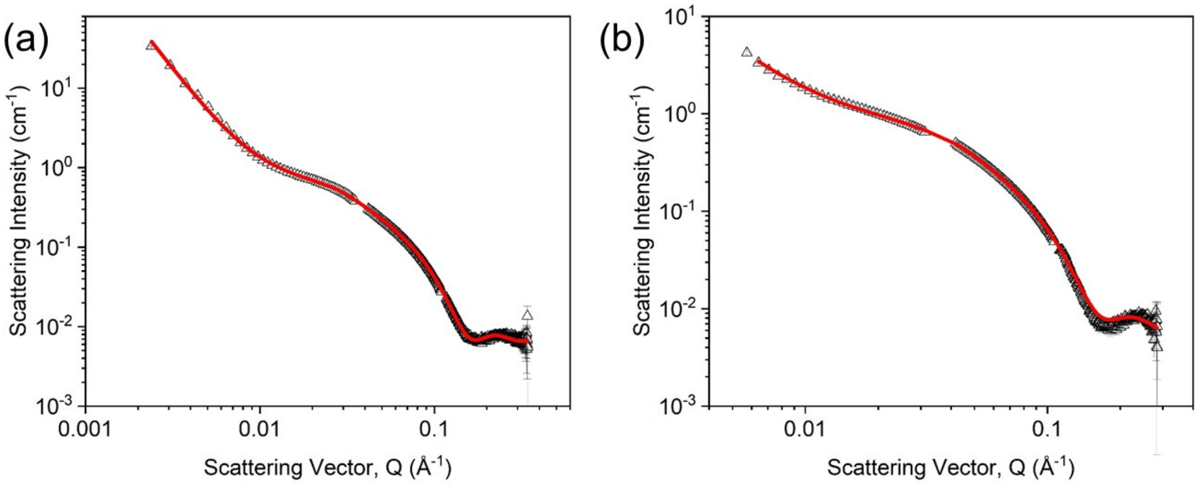

Figure S14. SAXS data (black hollow triangles) and fits (red lines) from the vertical bow-tie integrations of the 1ThNapFF pH series at 10 mg/mL with (a) pH 9.2, (b) pH 12.0.

Table S2. Processing type and fitting parameters for 10 mg/mL 1ThNapFF at pH 9.2 and 12.0.

**pH**	**9.2**	**12.0**
**Processing type**	Vertical bow-tie integration	Vertical bow-tie integration
**Fitting model**	Flexible cylinder + power law	Flexible cylinder + power law
**Cylinder scale**	0.001380	0.002010
**Cylinder scale error**	0.000002	0.000005
**Background**	0.0066	0.005*
**Background error**	0.0000280	-
**Radius / Å**	22.3	22.0
**Radius error / Å**	0.02	0.04
**Length / Å**	167.4	132.7
**Length error / Å**	0.4	0.7
**Kuhn length / Å**	110.88	77.85
**Kuhn length error / Å**	0.36	0.41
**Power law scale**	0.00000138	0.00007636
**Power law scale error**	0.00000002	0.00000224
**Power law**	2.84	2.07
**Power law error**	0.0029445	0.0061121
**Reduced Chi^2**	11.46	9.40

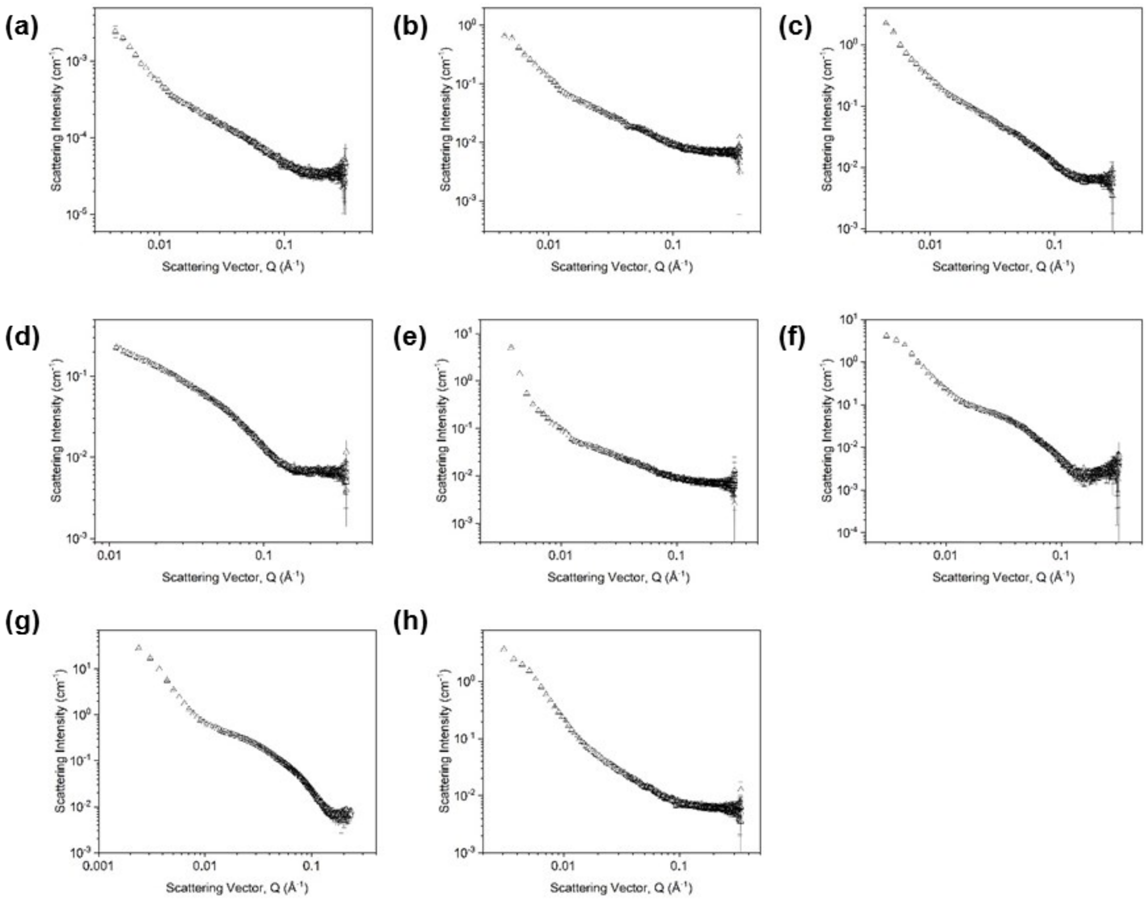

Figure S15. SAXS data for the horizontal (low scattering intensity) bow-tie integrations for the anisotropic data sets. (a) 2 mg/mL; (b) 2.5 mg/mL; (c) 3 mg/mL; (d) 4 mg/mL; (e) 5 mg/mL; (f) 15 mg/mL; (g) 10 mg/mL pH 9.2 and (h) 10 mg/mL pH 12.0. All samples at pH 11.3 unless stated otherwise.

When studying the minimum concentration at which noodles could be formed, 80 ppm Nile Blue A was added to the lower concentration solutions to better visualise the structures that formed upon injection into the trigger medium. A SAXS experiment was performed to check the influence of this additive to the self-assembly (Figure S16 and Tables S3 and S4). The SAXS data for 2 mg/mL 1ThNapFF and 2 mg/mL 1ThNapFF with 80 ppm Nile Blue A both fit to cylinder models with radii of approximately 2.1 nm. With the addition of the Nile Blue A, the data requires a flexible component in the model to be fitted. Nevertheless, with or without Nile Blue A the data sets depict very similar structures.

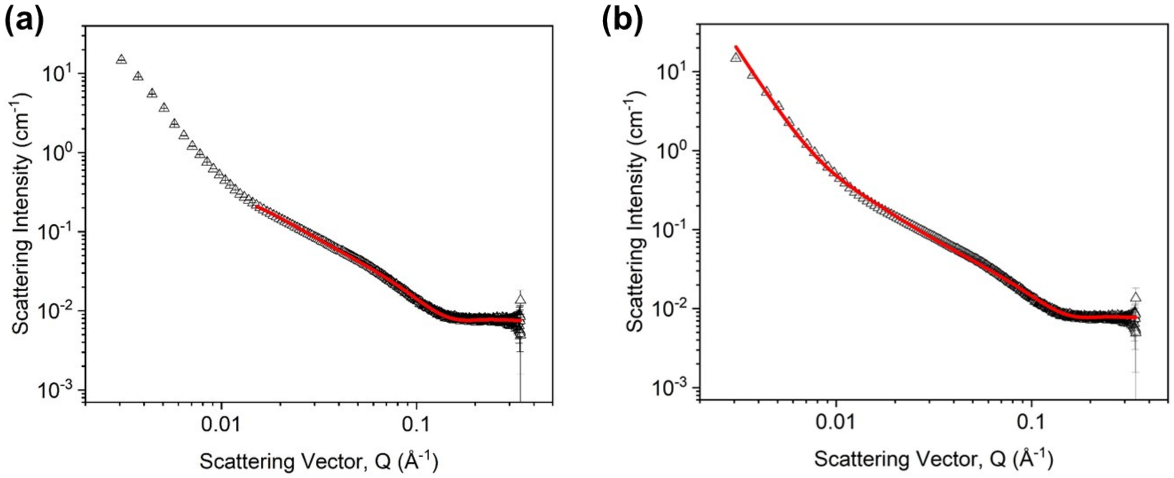

Figure S16. SAXS data for 2.0 mg/mL 1ThNapFF with 80 ppm Nile Blue A additive with corresponding models fits at (a) a reduced q range and (b) the whole q range.

Table S3. Flexible cylinder fit parameters obtained for 2.0 mg/mL 1ThNapFF with 80 ppm Nile blue A with the fit shown in Figure S16 a.

Scale	Scale Error	Background	Background Error	Length / Å	Length error / Å	Kuhn Length / Å	Kuhn Length Error / Å	Radius / Å	Radius Error / Å	Scattering length density / x10^{-6} Å^{-2}	Solvent SLD / x10^{-6} Å^{-2}	Reduced Chi^2
0.000252	0.00000149	0.007533	0.0000264	536.86	9.3382	134.21	0.000776	20.596	0.066938	14.025	9.469	1.3

Table S4. Flexible cylinder combined with a power law Fit parameters obtained for 2.0 mg/mL 1ThNapFF with 80 ppm Nile blue A with the fit shown in Figure S17 b.

Scale	Background	Background Error	Flexible Cylinder scale	Flexible Cylinder Error	Length / Å	Length error / Å	Kuhn length / Å	Kuhn length error / Å	Radius / Å	Radius error / Å	Scattering length density / x10^{-6} Å^{-2}	Solvent SLD / x10^{-6} Å^{-2}	Power law scale	Power law scale error	Power law	Power law error	Reduced Chi^2
1	0.007889	2.64x10^{-5}	0.000229	1.49x10^{-6}	536.86	7.785	134.21	0.000634	20.596	0.074075	14.025	9.469	4.86x10^{-9}	3.01x10^{-10}	3.8255	0.011323	19.1

2.3 Pipette filament formation

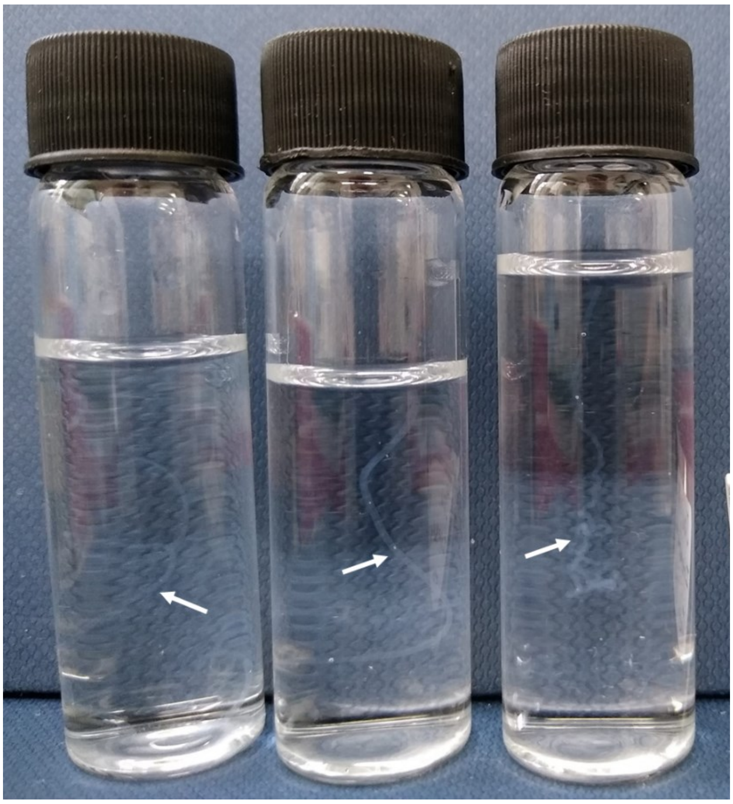

Figure S17. Photograph of gel filaments formed using a 2-20 μL pipette at slow (left), intermediate (middle) and fast (right) injection speeds. White arrows have been added to guide the eye to filaments.

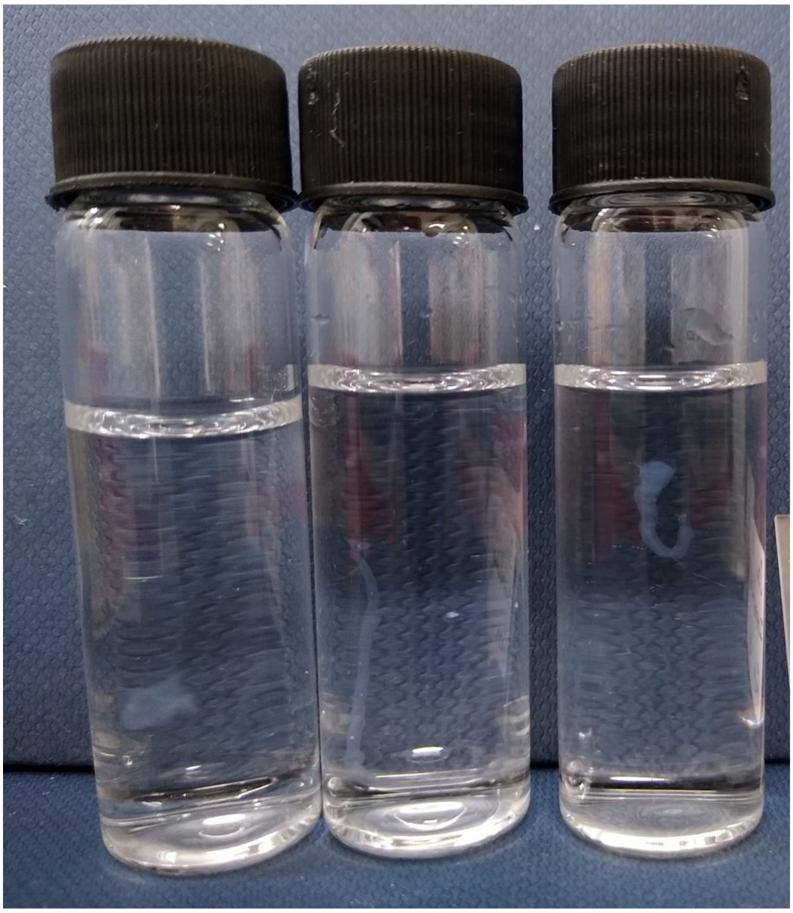

Figure S18. Photograph of gel filaments formed using a 20-200 μL pipette at slow (left), intermediate (middle) and fast (right) injection speeds

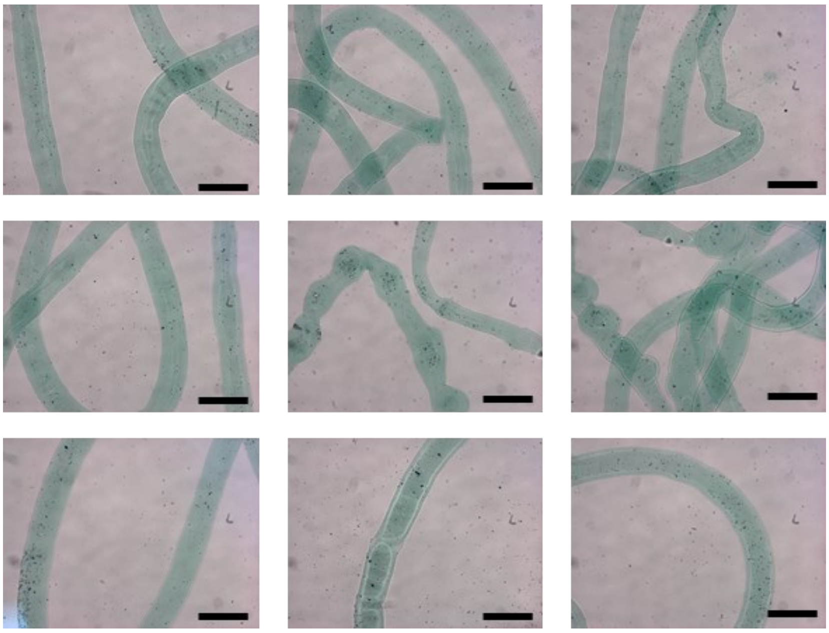

Figure S19. Optical microscope images of gel filaments made with 2 mg/mL 1ThNapFF with 80 ppm Nile Blue A. Scale bars represent 0.6 mm.

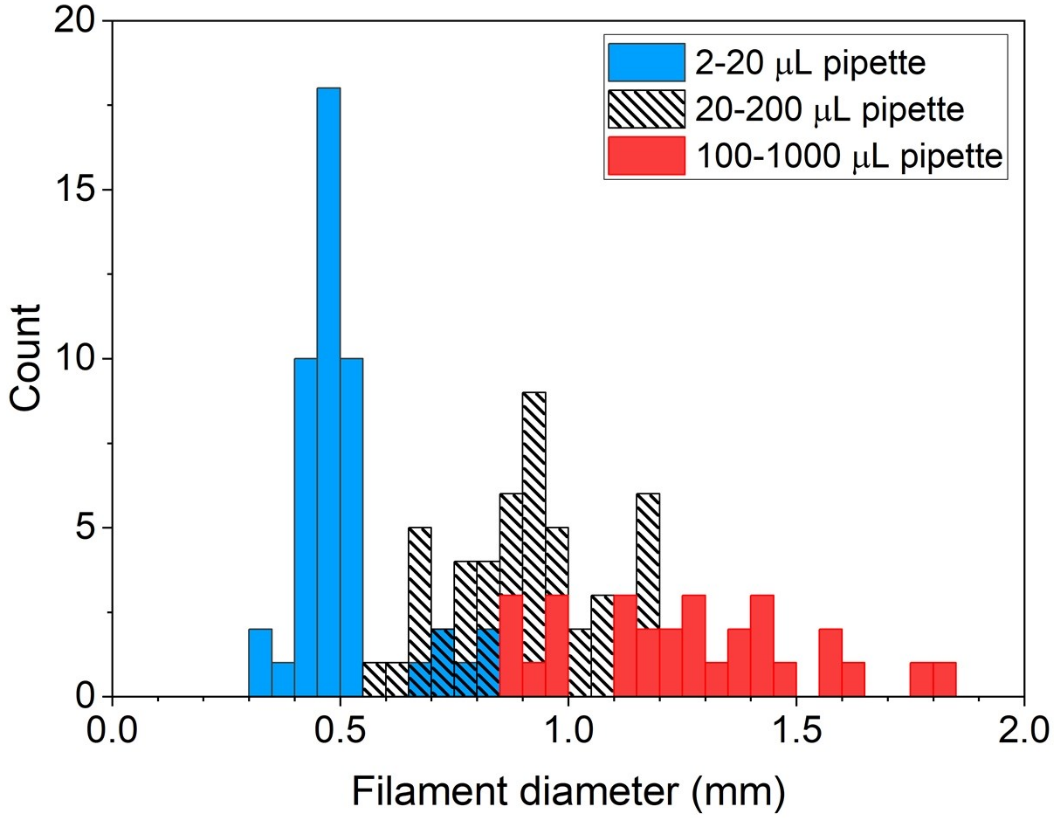

Figure S20. Histogram of filament diameters formed using three types of pipette.

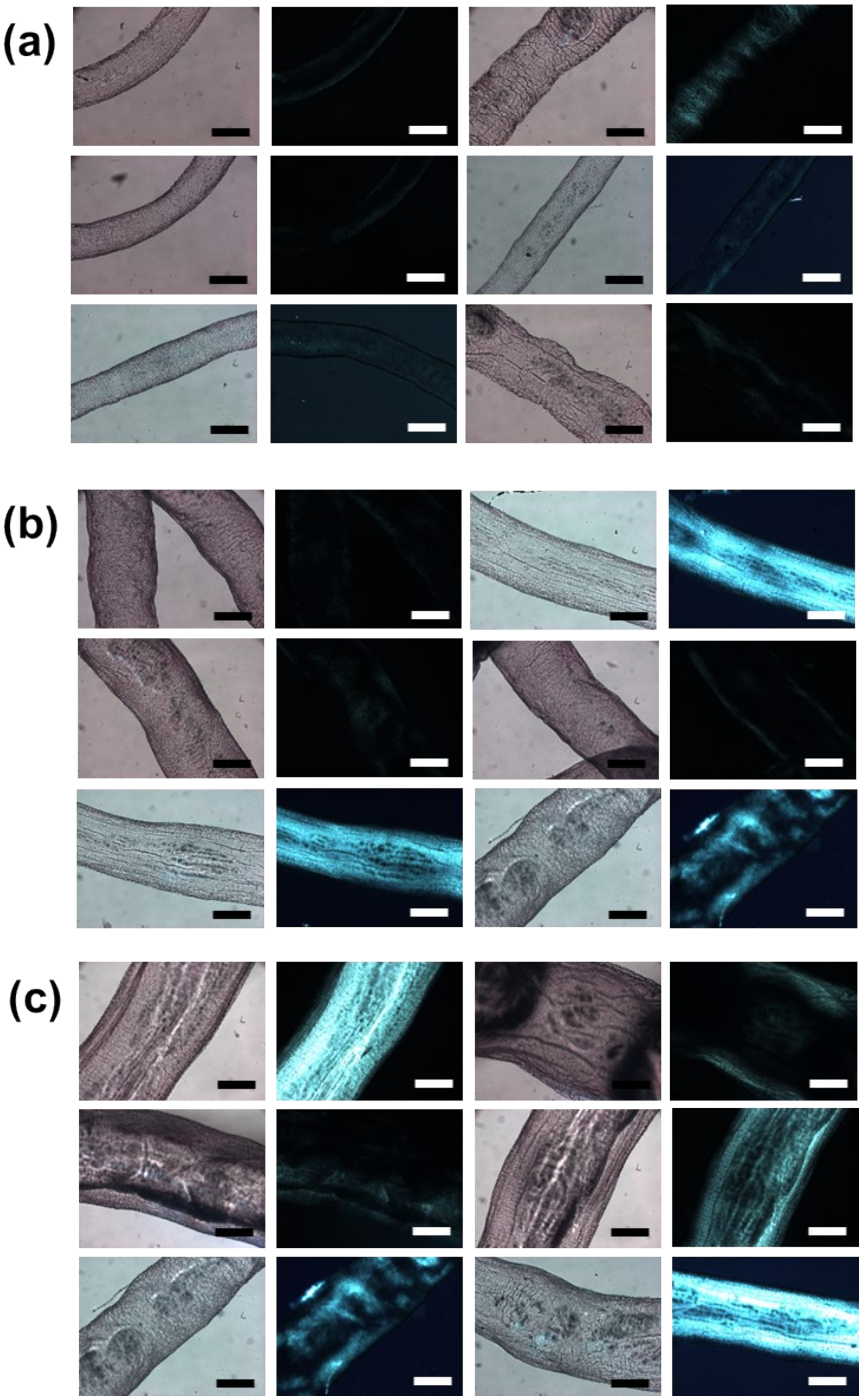

Figure S21. Microscope images of gel filaments formed from (a) a 2-20 μL pipette; (b) a 20-200 μL pipette and (c) a 100-1000 μL pipette under static conditions. Two images are given for each filament region that was imaged. One under normal light and the other under cross polarised light. In each set of images the left hand images are under normal light, the right images are under cross polarised light. Scale bars represent 0.5 mm.

Filaments were formed whilst dragging the pipette through trigger medium. This technique was challenging to control and resulted in a large variation in filament diameters however the thin, stretched regions typically showed birefringence. Due to the irreproducibility of these filaments, a mean filament diameter was not measured.

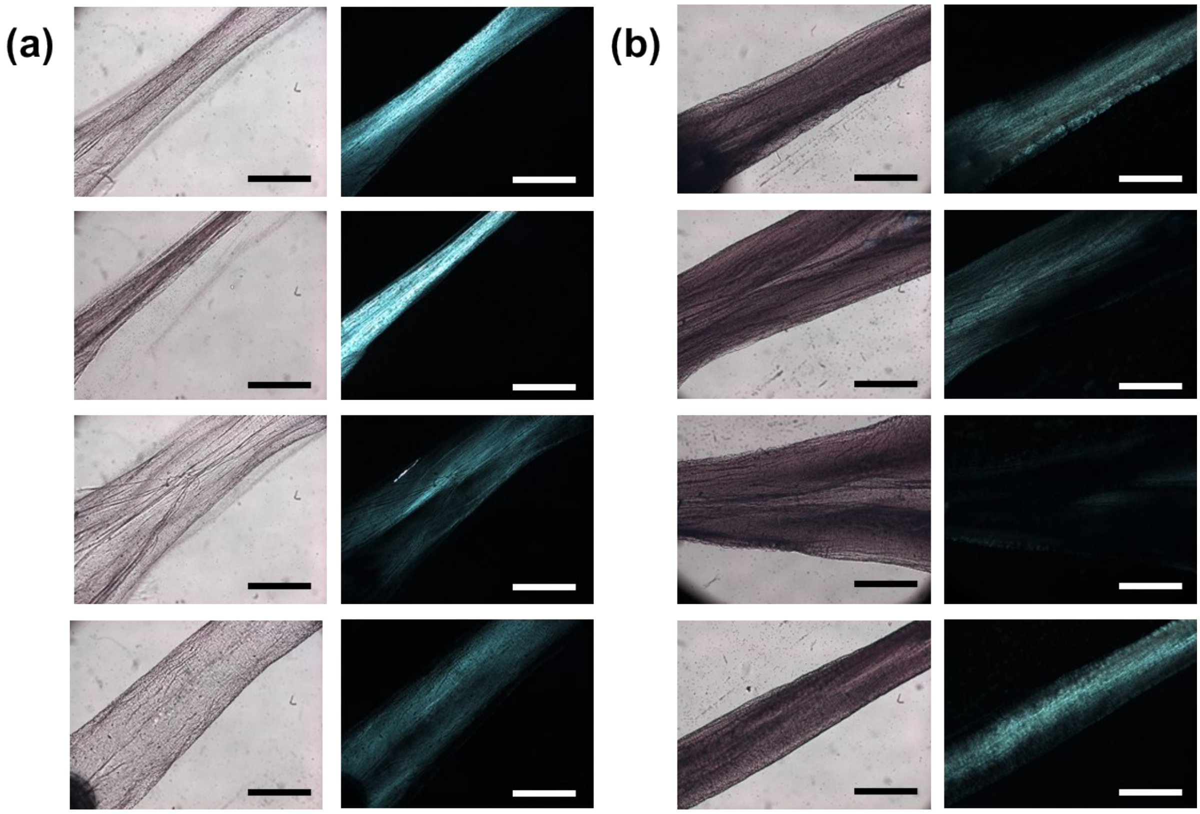

Figure S22. Microscope images of dragged gel filaments formed from (a) a 20-200 µL pipette and (b) a 100-1000 µL (1 mL) pipette. In each set of images the left hand images are under normal light, the right images are under cross polarised light. Scale bars represent 0.6 mm.

2.4 Syringe pump filament formation

A photograph of some gel filaments formed using a syringe pump and needle are shown below (Figure S23).

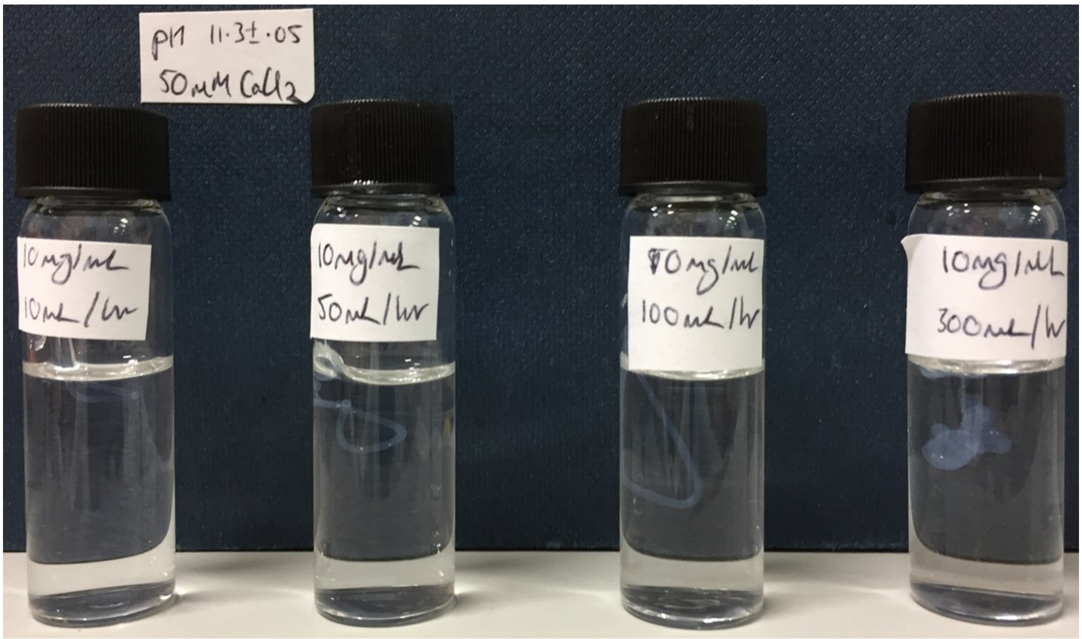

Figure S23. Photograph of gel filaments formed in bulk trigger medium using different flow rates. The sample labelled “300 mL/hr” can be seen to have formed very thick and inhomogeneous gel filaments, highlighting the need for an optimised flow rate.

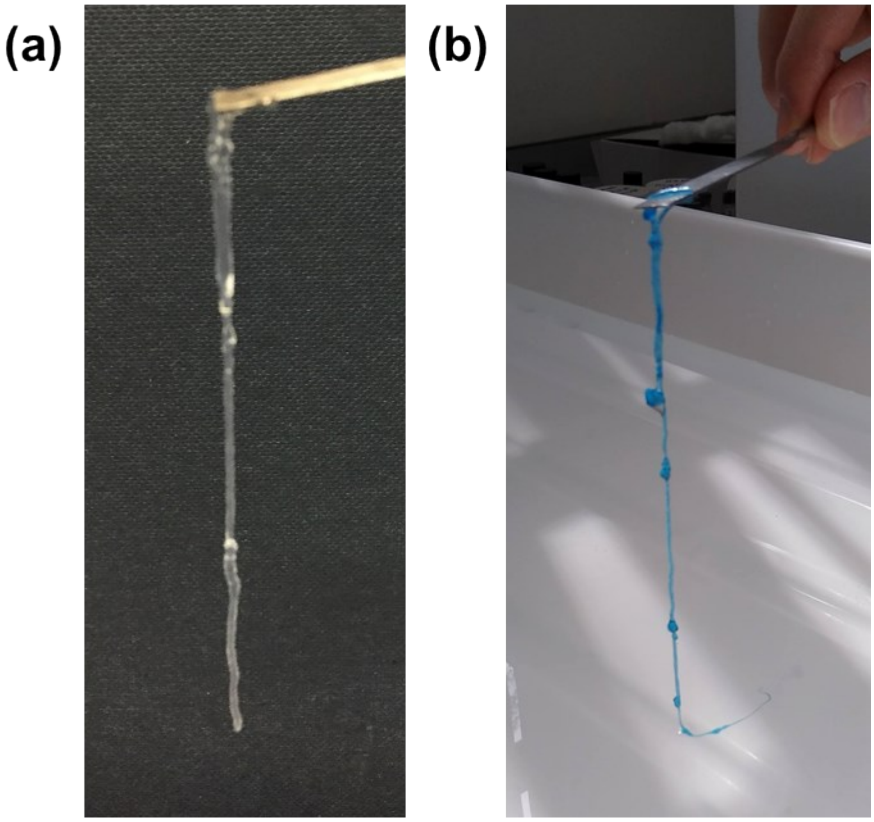

Figure S24. Photograph of (a) an approximately 5 cm 10 mg/mL 1ThNapFF gel filament and (b) a 5 mg/mL 1ThNapFF (Nile blue A stained) gel filament. Both have been lifted out of the trigger medium

on a spatula. They are the result of lifting a filament at the middle to balance around the spatula, such the images show two filaments hanging next to each other.

2.4.1 Effect of flow rate under static formation

With the syringe pump, the filament diameter could be controlled by changing the flow rate. This allows for fine tuning of the structures formed.

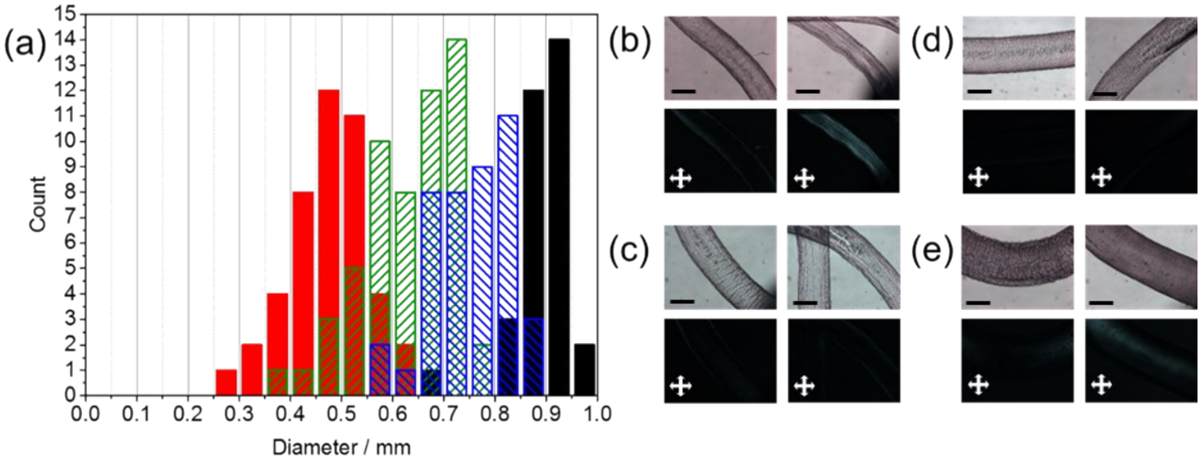

Figure S25. (a) Histogram of the filament sizes formed at flow rates of 10 mL/hr (solid red), 25 mL/hr (dashed green), 50 mL/hr (dashed blue) and 100 mL/hr (solid black); microscope images for (b) 10 mL/hr; (c) 25 mL/hr; (d) 50 mL/hr; (e) 100 mL/hr. Scale bars represent 0.5 mm.

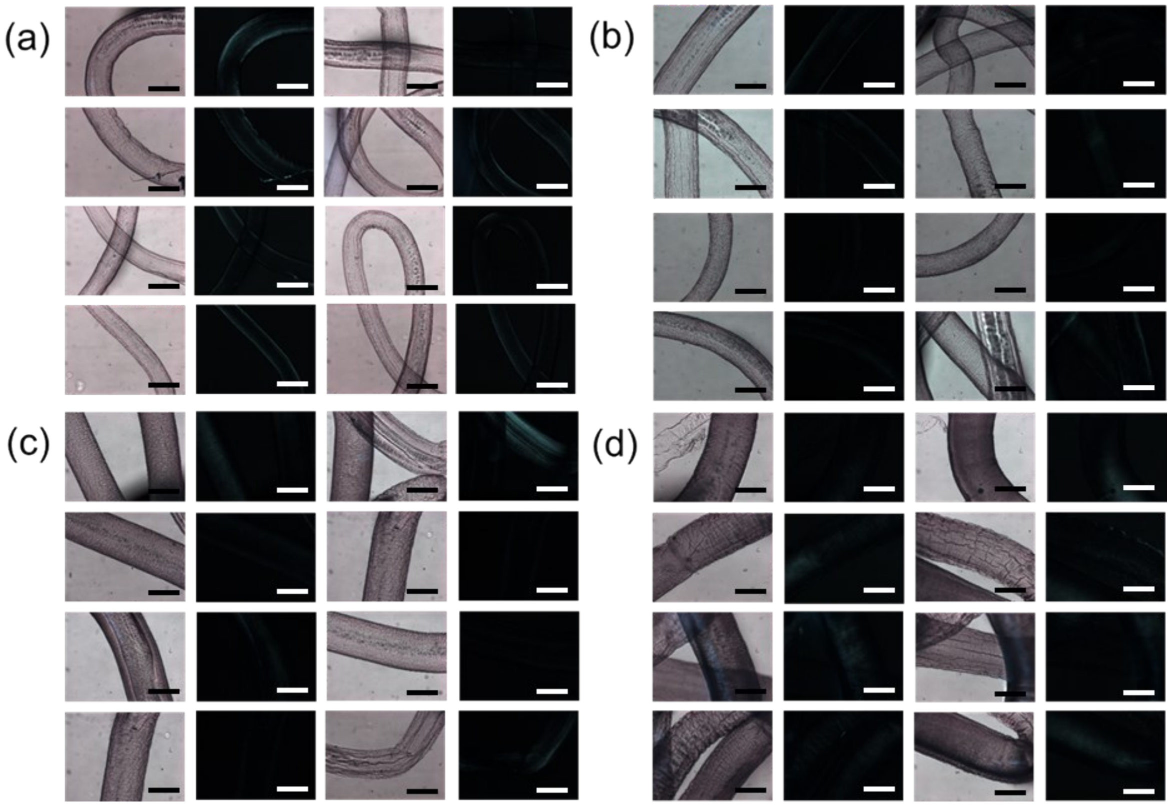

Figure S26. A selection of representative optical microscope images (both normal and cross polarised light) to compliment the data in Figure S25. The gel structures are formed from 10 mg/mL 1ThNapFF at pH 11.3 using the static procedure investigating the effect of flow rate. (a) 10 mL/hr; (b) 25 mL/hr; (c) 50 mL/hr and (d) 100 mL/hr. Scale bars represent 0.6 mm.

2.4.2 Effect of 1ThNapFF concentration under static formation

The effect of 1ThNapFF concentration was investigated. The solution concentration influenced the filament diameter at 10 mL/hr, with the lower concentrations forming thinner filaments. These differences are attributed to the difference shear viscosities of the solutions at difference concentrations. The SAXS data shows similar structures are present, but it is hypothesised that these differences are due to other factors (such as the concentration of self-assembled structures present) not determined from the SAXS analysis. At 100 mL/hr the noodles formed are the same diameter.

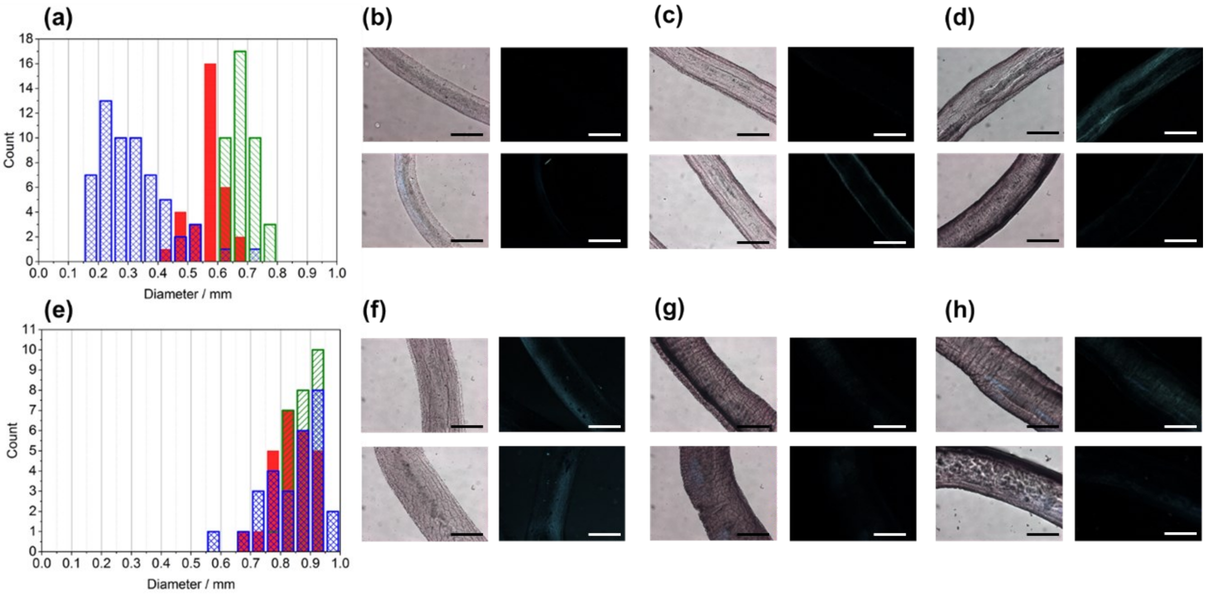

Figure S27. (a) Histogram of filament diameters for 5 mg/mL (blue, crisscrossed), 10 mg/mL (red, solid) and 15 mg/mL (green, dashed) 1ThNapFF at a flow rate 10 mL/hr with corresponding optical microscope images both normal and cross polarised light for (b) 5 mg/mL; (c) 10 mg/mL and (d) 15 mg/mL; (e) Histogram of filament diameters for 5 mg/mL (blue, criss crossed), 10 mg/mL (red, solid) and 15 mg/mL (green, dashed) 1ThNapFF at a flow rate 100 mL/hr with corresponding optical microscope images both normal and cross polarised light for (f) 5 mg/mL; (g) 10 mg/mL and (h) 15 mg/mL. Scale bars represent 0.6 mm.

2.4.3 Effect of solution pH

The effect of 1ThNapFF solution pH was investigated. The pH will influence the surface charge on the worm-like micelles and consequently could affect the interactions between them.

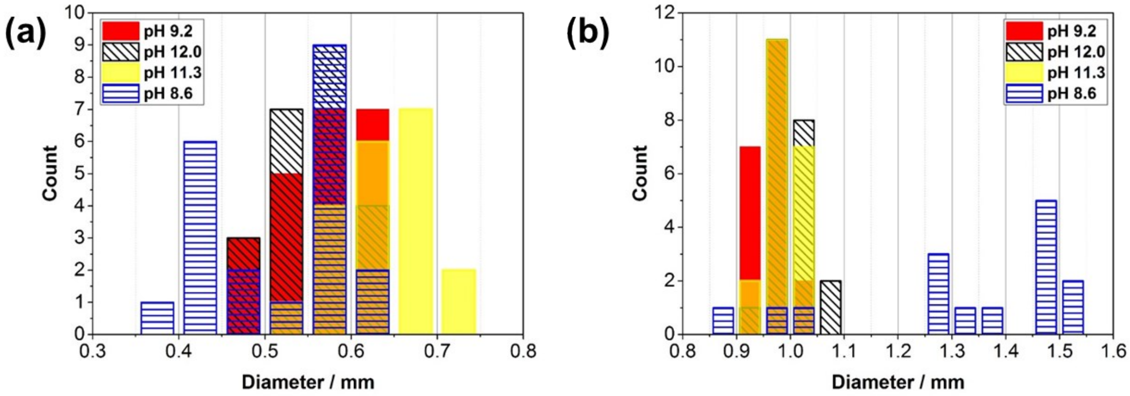

Figure S28. Histograms of filament diameter formed from 10 mg/mL 1ThNapFF at different pH at both (a) 10 mL/hr and (b) 100 mL/hr.

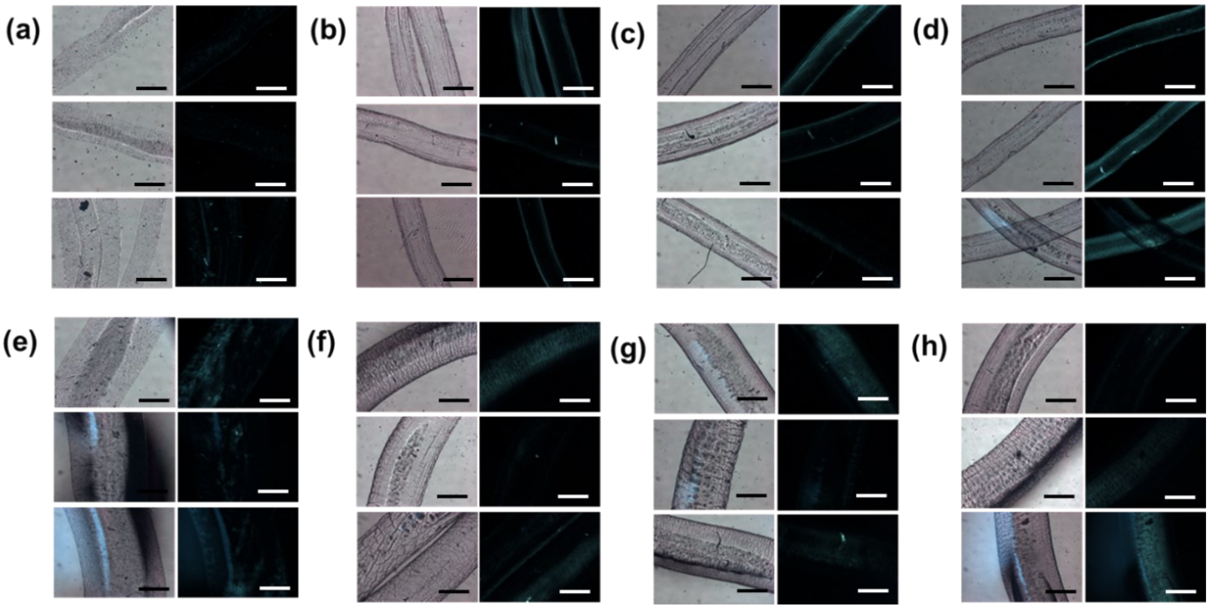

Figure S29. Microscope images of 1ThNapFF filaments formed with 10 mg/mL at different pH. Top row (a – d) represent filaments formed at 10 mL/hr at (a) pH 8.6; (b) pH 9.2; (c) pH 11.3 and (d) pH 12.0. In each pair of images the left hand ones are under normal light and the right hand ones under cross polarised light. Scale bars represent 0.6 mm.

2.4.4 Multi-meter long continuous filaments

With the syringe pump setup multi-meter long gel filaments can be formed. The only limitation is the size of the pre-gel solution reservoir (syringe) and the size of the trigger medium bath.

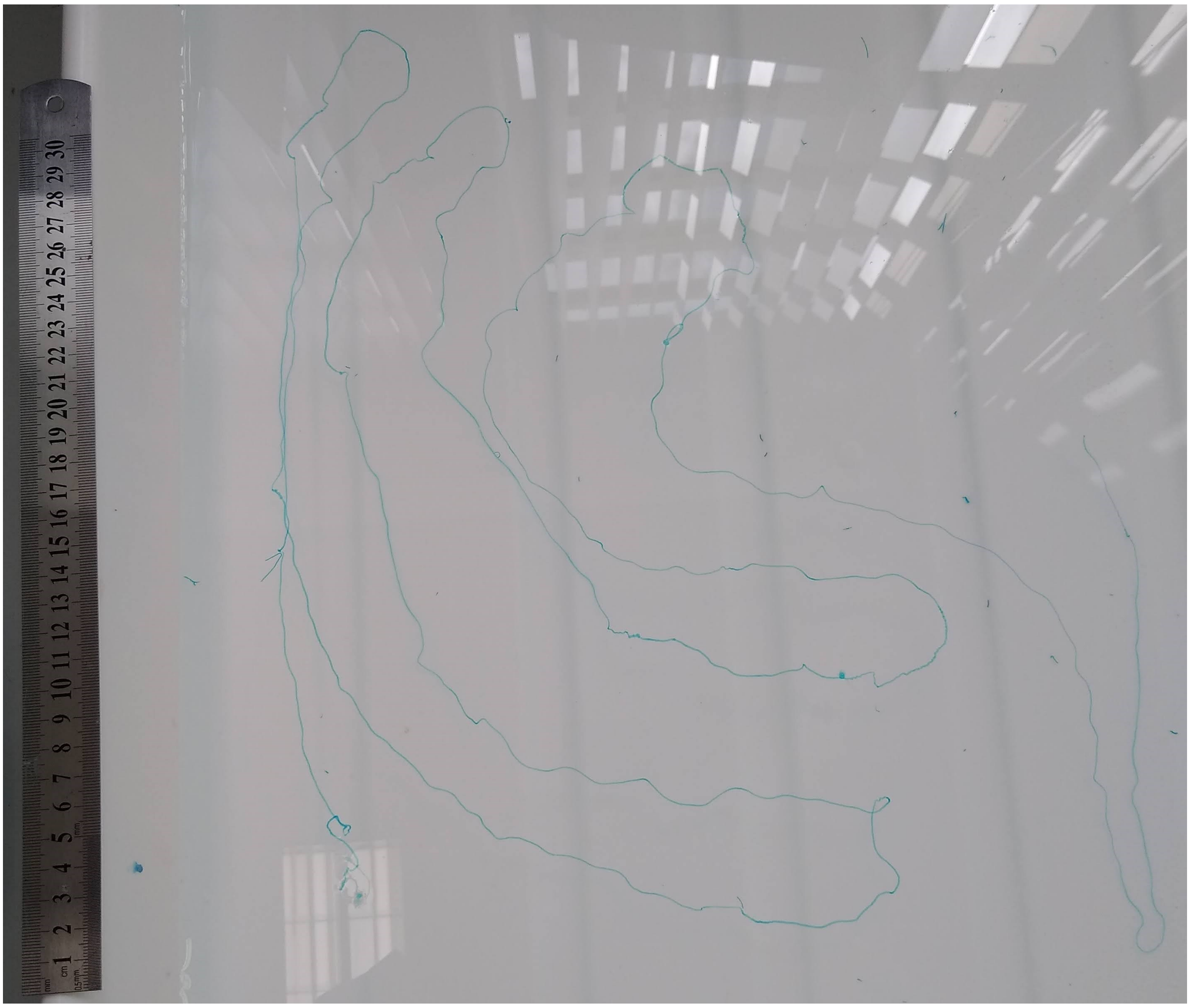

Figure S30. The full photograph of a multi-meter continuous gel filament (presented in the main paper) formed from a 10 mg/mL 1ThNapFF solution stained with 80 ppm Nile Blue A in a 50 mM $CaCl_2$ bath. A 30 cm ruler is placed to the left of the photograph for scale.

2.4.5 Results from the spinning technique

Using the spinning technique, four flow rates were investigated and three repeats performed for each flow rate. Aligned filaments were formed under every flow rate (Figure S32) and the size distribution was centred around 0.1 mm regardless of the flow rate (Figure S31).

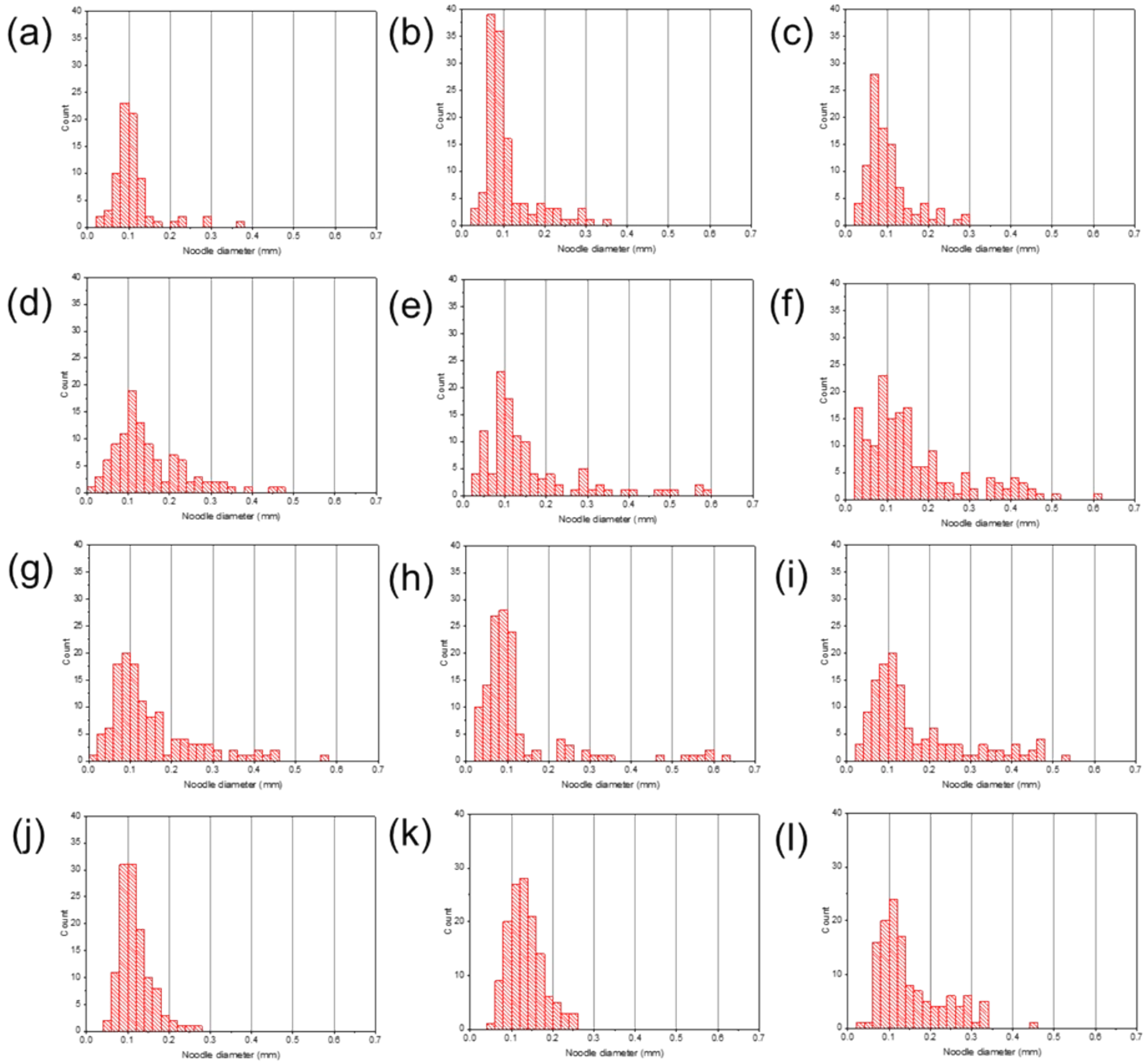

Figure S31. Histograms for the 100 rpm spinning technique. A 10 mg/mL 1ThNapFF pre-gel solution at pH 11.3 was used. (a – c) three repeats at 10 mL/hr; (d – f) three repeats at 25 mL/hr; (g – i) three repeats at 50 mL/hr and (j – l) three repeats at 100 mL/hr. Corresponding microscope images are shown in Figure S32.

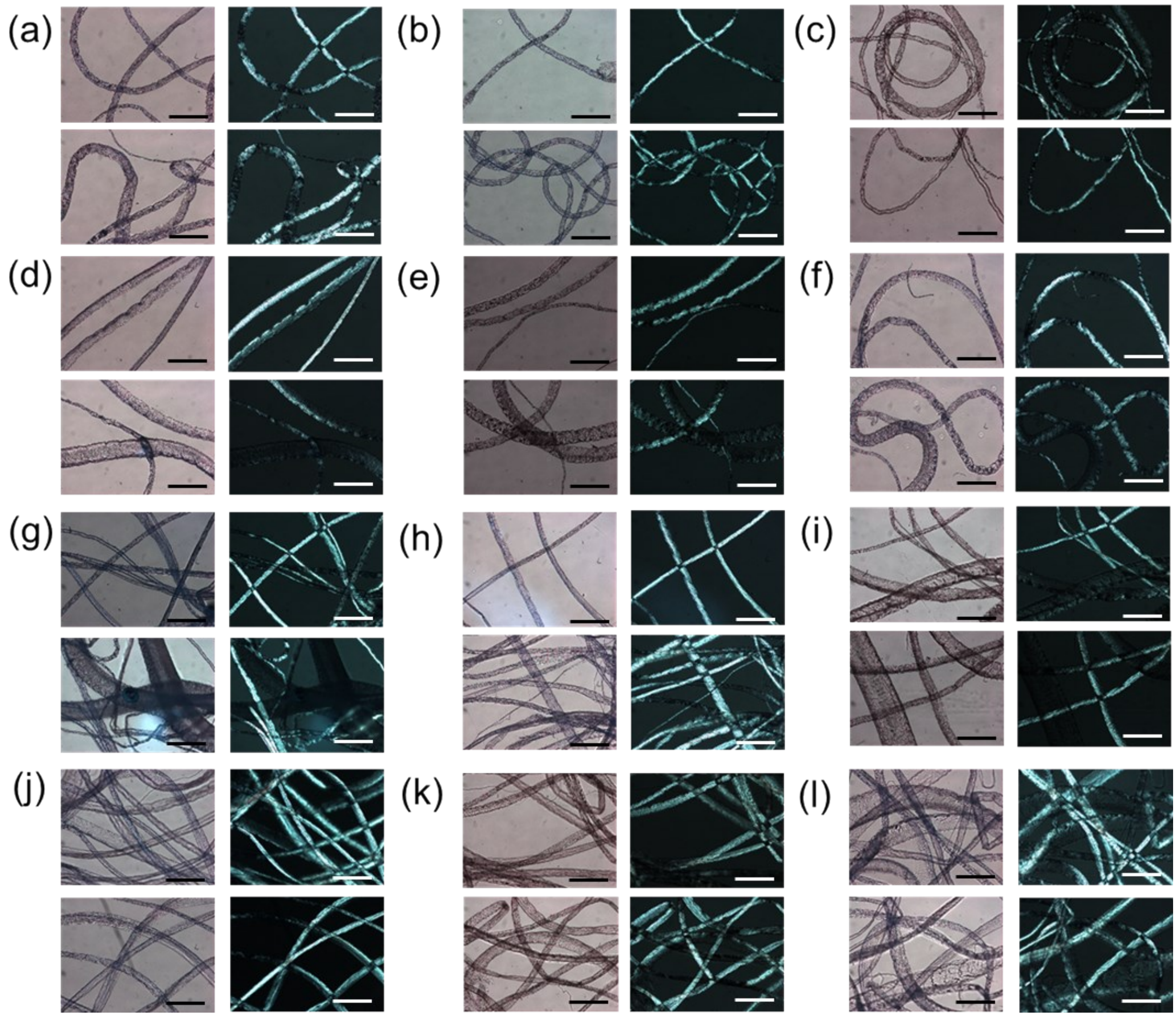

Figure S32. Corresponding optical microscope images (both normal and cross polarised light) for the 100 rpm spinning technique (histograms in Figure S31). A 10 mg/mL 1ThNapFF pre-gel solution at pH 11.3 was used. (a – c) three repeats at 10 mL/hr; (d – f) three repeats at 25 mL/hr; (g – i) three repeats at 50 mL/hr and (j – l) three repeats at 100 mL/hr. Scale bars represent 0.6 mm.

2.4.6 Effect of a heat/cool cycle

For 1ThNapFF, a heat/cool was not required to form aligned gel filaments. The influence of a heat/cool cycle was investigated with temperatures of both 60°C and 80°C. A 60°C heat/cool had little impact on the shear rheology of the 1ThNapFF solution (Figure S9) as well as the gel filaments that are formed (Figure S33b, S34b, S35b). The 80°C heat/cool resulted in solutions with an increased viscosity (Figure S9) but resulted in gel filaments with no alignment seen using CPOM (Figure S33c, S34c, S35c).

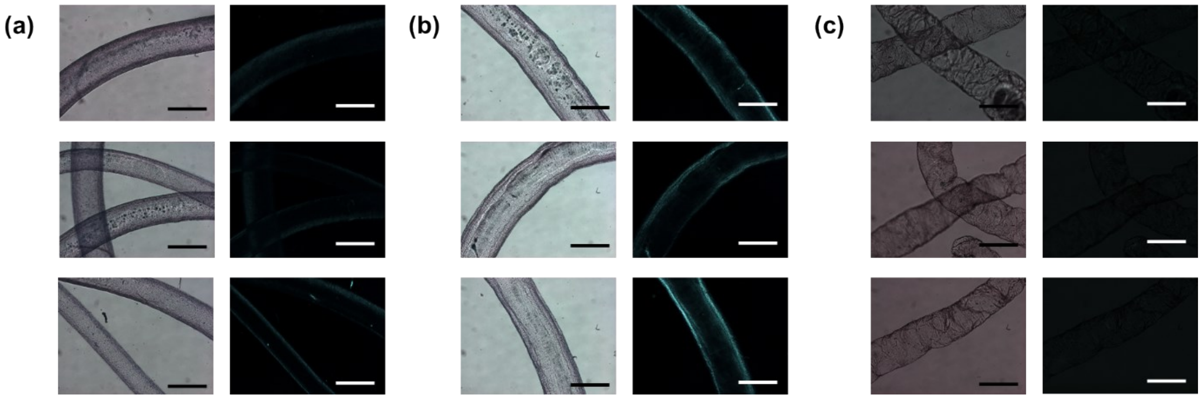

Figure S33. Microscope images of gel filaments made from 10 mg/mL 1ThNapFF at pH 11.3 and a flow rate of 10 mL/hr under static conditions. Pre-gel solutions (a) not heat/cooled; (b) heat/cooled to 60°C and (c) heat/cooled to 80°C were used to form the filaments. In (a), (b) and (c) the images on the left are under normal light and those on the right are under cross polarised light. Scale bars represent 0.6 mm.

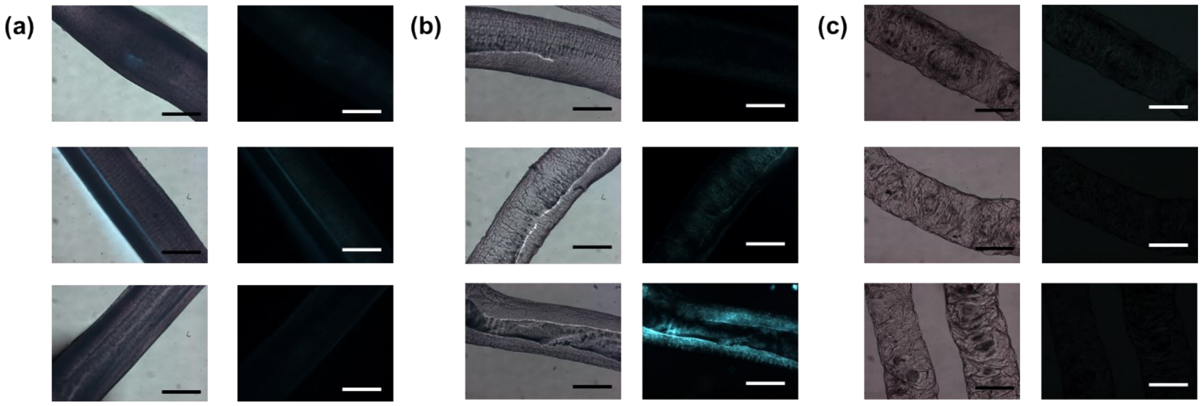

Figure S34. Microscope images of gel filaments made from 10 mg/mL 1ThNapFF at pH 11.3 and a flow rate of 100 mL/hr under static conditions. Pre-gel solutions (a) not heat/cooled; (b) heat/cooled to 60°C and (c) heat/cooled to 80°C were used to form the filaments. In (a), (b) and (c) the images on the left are under normal light and those on the right are under cross polarised light. All images are captured in transmission mode at 5X magnification. Scale bars represent 0.6 mm.

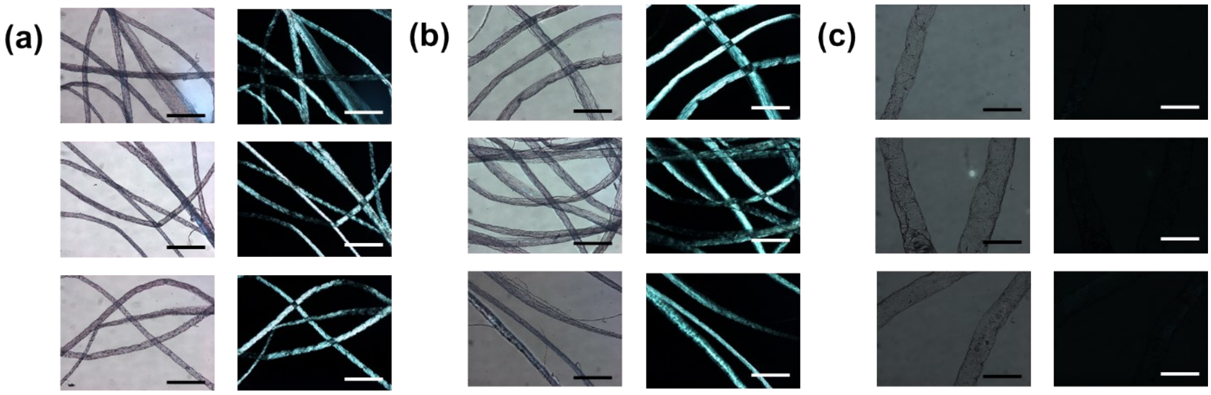

Figure S35. Microscope images of gel filaments made from 10 mg/mL 1ThNapFF at pH 11.3 and a flow rate of 100 mL/hr and the spinning technique. Pre-gel solutions (a) not heat/cooled; (b) heat/cooled to 60°C and (c) heat/cooled to 80°C were used to form the filaments. In (a), (b) and (c) the images on the left are under normal light and those on the right are under cross polarised light. All images are captured in transmission mode at 5X magnification. Scale bars represent 0.6 mm.

2.5 Nanoindentation

Nanoindentation was used to measure the mechanical properties of the gel filaments formed and bulk 1ThNapFF gels formed with $CaCl_2$. As a highly localised technique, a wide range of different values are obtained for each sample due to local differences in the mechanical strength. To best represent these data, violin plots were created. In the violin plots the white dots represent the median Young's modulus, the thick dark blue bars represent the 1st and 3rd quartiles, the thin dark blue lines represent all the data excluding outliers and the light blue coloured violin-shaped regions represent the whole distribution of the Young's moduli values for each data set. The thicker the light blue region, the more data is present around that Young's modulus value.

(a) (b) (c) (d)

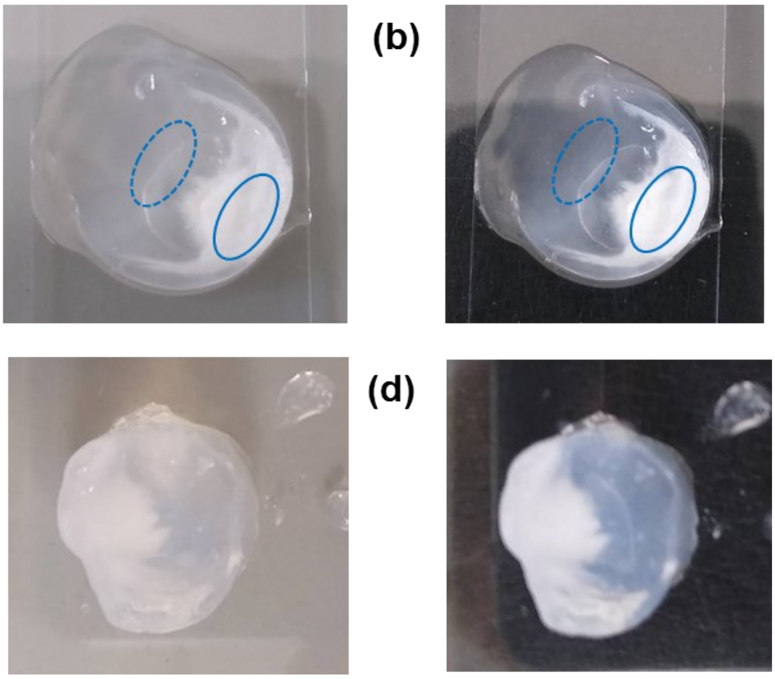

Figure S36. Photographs of the bulk gels studied with nanoindentation. A 7 day diffusion time bulk gel with a (a) white and (b) black background. For (a) and (b), the dotted blue circles represent the soft side and the solid line blue circles represent the stiff side of the inhomogeneous gel. A 6 month diffusion time bulk gel with a (c) white and (b) black background.

For the inhomogeneous gel, the transition from the stiff to the soft side can be clearly seen (Figure S36a and b) and at the extremes edge of the soft side, the sample is still a liquid. In the nanoindentation data the stiff side of the gel had significantly greater Young's modulus values than the softer side of the gel (Figure S37a). The median Young's modulus of the stiff side of the gel was approximately 130 kPa and the median value for the soft side was approximately 7 kPa. This highlights the importance of the Ca^{2+} concentration on the mechanical properties of the gel. The homogeneous 6 month old gel showed a median Young's modulus at around 15 kPa.

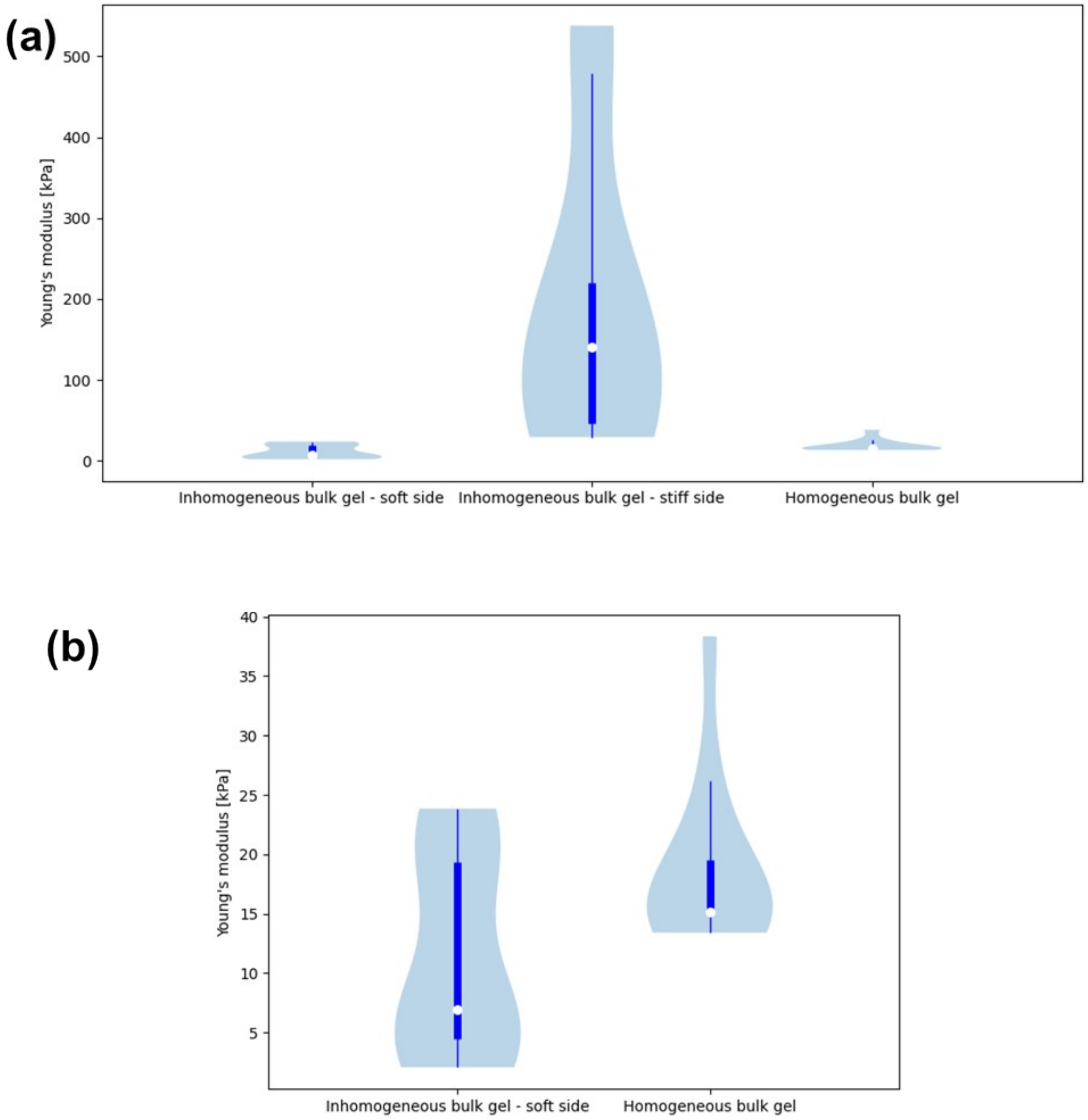

Figure S37. Violin plots of nanoindentation data for bulk gels of 1ThNapFF prepared with $CaCl_2$. (a) full scale plot with all three sets of data and (b) the same data replotted and excluding the data for the stiff part of the inhomogeneous bulk gel.

The effect of aging on the gel filaments was investigated (Figure S38). The aging was only studied over a 24 hours period but showed that the Young's modulus fell over 24 hours from around 5 kPa to 2 kPa.

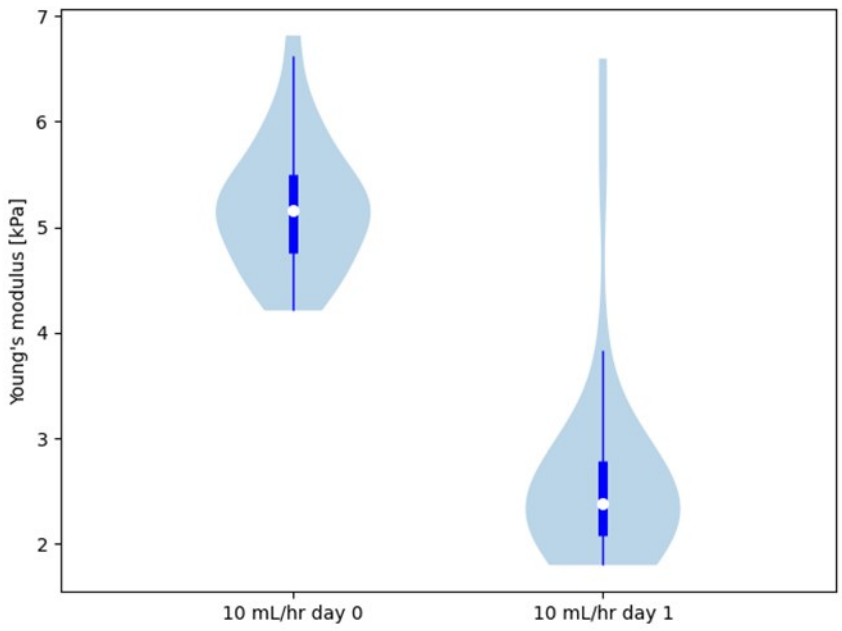

Figure S38. Violin plots for a gel filament formed with a syringe pump at a flow rate of 10 mL/hr on the day it was made (day 0) and 24 hours later (day 1).

A cross-section of a filament-in-filament was cut using a scalpel blade and the mechanical properties of the two regions probed using nanoindentation (Figure S39). For this sample, the Young's moduli of the two regions are similar and most of the data overlap.

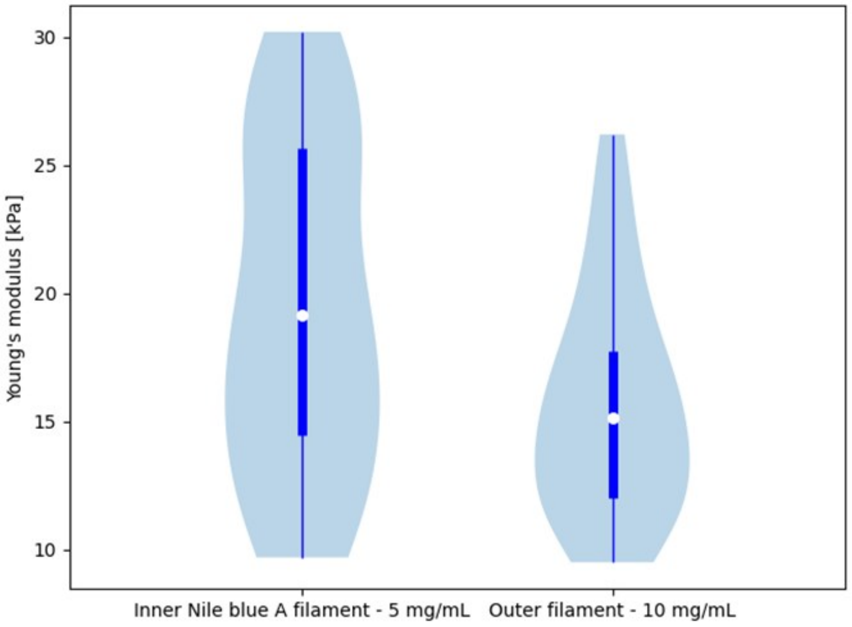

Figure S39. Violin plots of data collected from the cross-section of a filament-in-filament, allowing both the 10 mg/mL 1ThNapFF and 5 mg/mL 1ThNapFF (Nile blue A stained) regions to be measured

2.6 Concentric flow experiments

2.6.1 Aligned filament formation

It was hypothesised that a sufficiently fast sheath flow of 50 mM $CaCl_2$ would stretch and align the gel filaments formed from a 10 mg/mL 1ThNapFF solution inner flow. With the flow rates accessible with the syringe pumps, a fast enough 50 mM $CaCl_2$ sheath flow could not be achieved to significantly stretch the gel filaments as they formed from the inner flow. Instead, the inner flow was started with no sheath flow and a length of filament formed (Figure S40a). Then the sheath flow syringe was rapidly driven by hand, resulting in a significant thinning and enhanced birefringence of the filaments being formed (Figure S40b). All of the filament regions formed were collected in the glass dish containing 50 mM $CaCl_2$ and the filaments taken for imaging. These data reinforce the conclusion that a stretching process results in enhanced alignment within the gel structures. This could, in theory, be used to form very long aligned filaments, with the limiting factors being the volume of the syringes used.

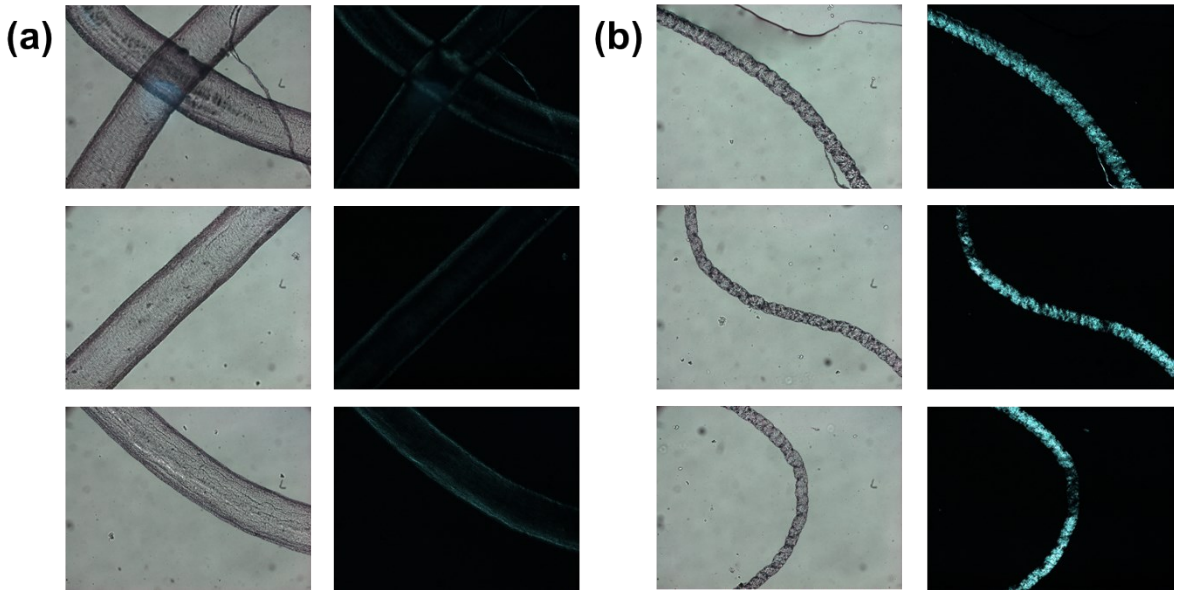

Figure S40. Gel filaments formed with the concentric flow setup with (a) no sheath flow and (b) a rapid, manually driven, sheath flow.

2.6.2 Filament-in-filament morphology

Using the concentric flow setup a filament-in-filament could be formed. The below flow rates were used to create two filament-in-filaments with differing widths of the inner Nile blue A stained gels. Corresponding photographs of these are shown below.

Filament-in-filament **1** (presented in main paper text)

Inner flow: 3 mL/hr

Sheath flow: 20 mL/hr

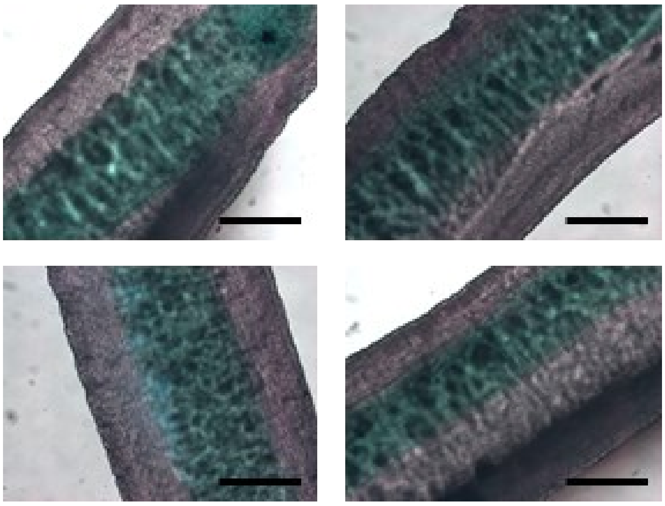

Figure S41. Additional optical microscope images of filament-in-filament **1** with an inner flow of 3 mL/hr and sheath flow of 20 mL/hr. The scale bars represent 0.6 mm.

<u>Filament-in-filament **2** (Figure S42 and S43)</u>

Inner flow: 10 mL/hr

Sheath flow: 50 mL/hr

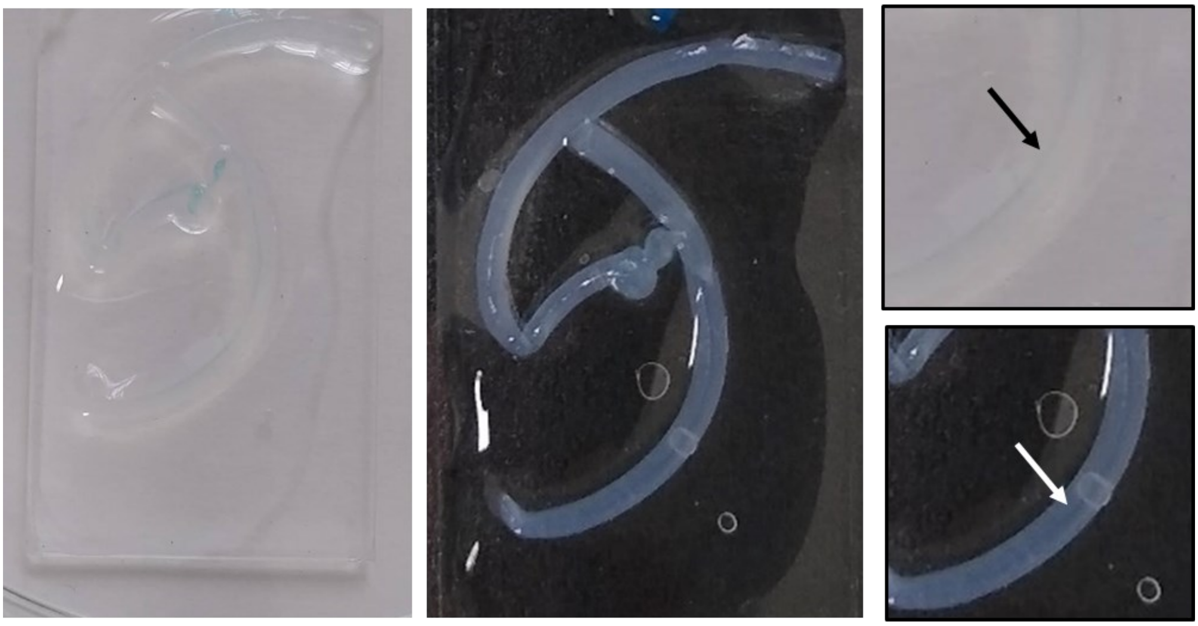

Figure S42. Photographs of filament-in-filament **2** with a white and a black background with two expanded images on the right with arrows to guide the eye to the thin blue inner filament.

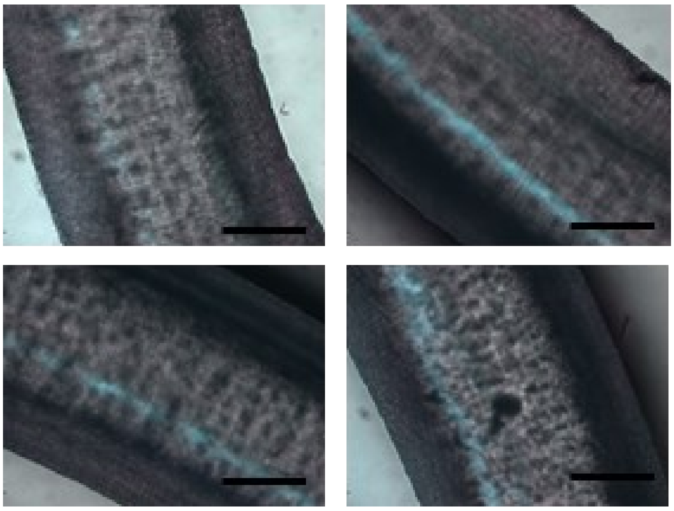

Figure S43. Optical microscope images of filament in filament **2**. For this sample, the inner blue filament cannot be seen in the microscope images. The blue shine on the surface of the filaments is an effect of the light. The scale bars represent 0.6 mm.

<u>Filament-in-filament **3** (Figure S44)</u>

Inner flow: 30 mL/hr

Sheath flow: 50 mL/hr

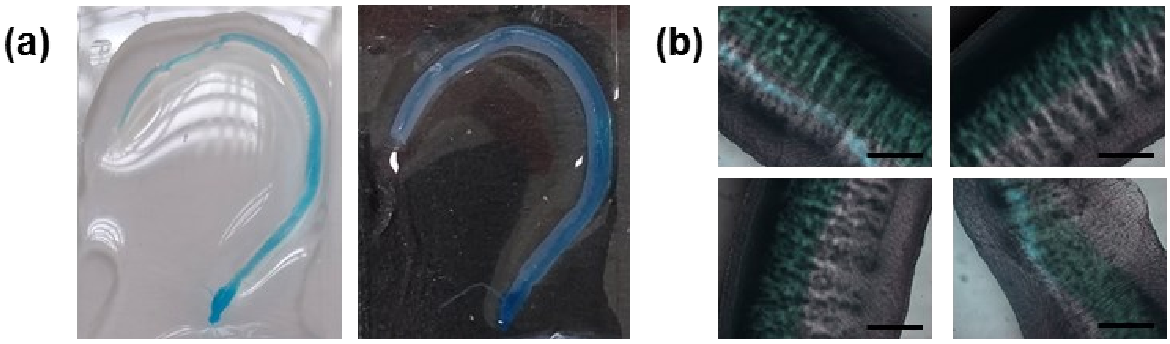

Figure S44. (a) Photographs of filament-in-filament **3** with a white and a black background. (b) Optical microscope images of filament in filament **3**. In (b), scale bars represent 0.6 mm.

3 References

1 M. C. Nolan, A. M. Fuentes Caparrós, B. Dietrich, M. Barrow, E. R. Cross, M. Bleuel, S. M. King and D. J. Adams, *Soft Matter*, 2017, **13**, 8426–8432.

2 L. Thomson, R. Schweins, E. R. Draper and D. J. Adams, *Macromol. Rapid Commun.*, , DOI:10.1002/marc.202000093.

3 S. Zhang, M. A. Greenfield, A. Mata, L. C. Palmer, R. Bitton, J. R. Mantei, C. Aparicio, M. O. De La Cruz and S. I. Stupp, *Nat. Mater.*, 2010, **9**, 594–601.

4 ImageJ, https://imagej.net/Welcome, (accessed 20 October 2019).

5 C. T. McKee, J. A. Last, P. Russell and C. J. Murphy, *Tissue Eng. - Part B Rev.*, 2011, **17**, 155–164.

6 I. Lüchtefeld, A. Bartolozzi, J. Mejía Morales, O. Dobre, M. Basso, T. Zambelli and M. Vassalli, *J. Nanobiotechnology*, 2020, **18**, 1–11.

7 M. Vassalli and G. Ciccone, CellMechLab/nanoindentation: beta, https://zenodo.org/record/4508646/export/json#.YMpon9VKiCo, (accessed 16 June 2021).

8 N. Gavara, *Sci. Rep.*, 2016, **6**, 1–13.

9 N. P. Cowieson, C. J. C. Edwards-Gayle, K. Inoue, N. S. Khunti, J. Doutch, E. Williams, S. Daniels, G. Preece, N. A. Krumpa, J. P. Sutter, A. D. Tully, N. J. Terrill and R. P. Rambo, *J. Synchrotron Radiat.*, 2020, **27**, 1438–1446.

10 Dawn Science, https://dawnsci.org/, (accessed 21 December 2020).

11 SasView, https://www.sasview.org/, (accessed 20 April 2020).

12 R. P. Murphy, Z. W. Riedel, M. A. Nakatani, P. F. Salipante, J. S. Weston, S. D. Hudson and K. M. Weigandt, *Soft Matter*, 2020, **16**, 6285–6293.

13 NIST Neutron activation and scattering calculator, https://www.ncnr.nist.gov/resources/activation/.

14 D. McDowall, B. Greeves, K. McAulay, A. Fuentes-Caparros, L. Thomson, N. Khunti, N. Cowieson, M. Nolan, M. Wallace, A. Cooper, E. Draper, A. Cowan, D. Adams and R. Clowes, *Adv. Energy Mater.*, 2020, **2002469**, 1–10.

15 E. R. Draper, H. Su, C. Brasnett, R. J. Poole, S. Rogers, H. Cui, A. Seddon and D. J. Adams, *Angew. Chem. Int. Ed.*, 2017, **56**, 10467–10470.

16 A. Z. Cardoso, L. L. E. Mears, B. N. Cattoz, P. C. Griffiths, R. Schweins and D. J. Adams, *Soft Matter*, 2016, **12**, 3612–3621.
